# Free Vibration of Porous Functionally Graded Graphene-Reinforced Cylindrical Shells on a Visco-Pasternak Foundation with General Boundary Conditions

**DOI:** 10.3390/ma19194251

**Published:** 2026-10-08

**Authors:** Hong Mei, Yunying Zhou, Yipei Meng, Yulong Wang, Xiangheng Zhang

**Affiliations:** 1 School of Architectural Engineering, North China Institute of Aerospace Engineering, Langfang 065000, China; 2 Hebei Key Laboratory of Trans-Media Aerial Underwater Vehicle, Langfang 065000, China; 3 School of Aeronautics and Astronautics, North China Institute of Aerospace Engineering, Langfang 065000, China

**Keywords:** visco-Pasternak foundation, complex boundaries, graded composition materials, circular cylindrical structures, dynamic response

## Abstract

This paper investigates the free vibration behavior of functionally graded graphene-reinforced porous cylindrical shells with complex boundary conditions, resting on a visco-Pasternak foundation, by utilizing the differential quadrature method (DQM). First, an improved Halpin–Tsai micromechanical model is employed to determine the effective material properties of the functionally graded graphene-reinforced porous cylindrical shells. Subsequently, based on the first-order shear deformation theory and Hamilton’s variational principle, the governing equations of motion are established. Finally, the differential quadrature method (DQM) is applied to obtain the reduced ordinary differential governing equations under various complex boundary conditions, and the accuracy of the numerical method is verified by comparison with the analytical solution for simply supported boundary conditions derived via trigonometric functions. In the numerical examples, the effects of boundary conditions, material parameters, geometric parameters, and visco-Pasternak foundation parameters on the vibration behavior of cylindrical shells are also discussed in detail.

## 1. Introduction

Functionally graded graphene-reinforced composites are widely used in the fields of aerospace, civil engineering, defense, and nuclear energy, due to their extremely high Young’s modulus, tensile strength, and excellent electrical, thermal, and mechanical properties [1,2,3,4]. By embedding graphene platelets into foamed metal [5], a functionally graded graphene platelet-reinforced porous composite (FG-GPLRPC) is formed. The composite combines the excellent mechanical properties of graphene-reinforced metals with the lightweight characteristics of porous materials. Also, it exhibits certain vibration-damping and energy-absorption properties [6], attracting widespread attention from researchers. The composite shell made of FG-GPLRPC material have broad application prospects in the fields of aerospace, civil engineering, ordnance, and nuclear energy [7,8]. Therefore, investigating the vibration behavior of functionally graded graphene-reinforced porous cylindrical shells is of great importance.

To date, a multitude of investigations into the mechanical behaviors of functionally graded graphene-reinforced composite (FG-GPLRC) structures have been carried out. For example, Hadji et al. [9] applied the third-order shear deformation theory to analyze the natural frequencies of non-ideal functionally graded laminated plates resting on a Winkler–Pasternak foundation. Based on the first-order shear deformation theory, Nosier et al. [10] investigated the nonlinear axisymmetric and nonaxisymmetric mechanical responses of functionally graded circular plates under transverse loading. Ye et al. [11] proposed a unified and exact method for solving the problem of free vibration of composite laminated thin shells with general elastic boundary conditions. Li et al. [12] developed a surface-similarity-scaled boundary element method based on a simulated plate-shell structure, and within the framework of the Hamiltonian system, obtained the surface stiffness matrix of the structure as well as the displacement and stress responses at an arbitrary point. Zuo et al. [13] used the first-order shear deformation theory and the Rayleigh–Ritz method to analyze the vibration of a functionally graded conical shell–annular plate composite structure under a temperature field. They examined the effects of parameters such as dimensions, material, temperature, and connection location on the structure’s vibration characteristics. Huang et al. [14] investigated the vibrational characteristics of porous graphene-reinforced truncated conical shells in an elastic medium based on Donnell’s classical thin-shell theory and Hamilton’s variational principle. Huang et al. [15] investigated the flutter and buckling characteristics of graphene-reinforced porous functionally graded truncated conical shells under high-speed airflow and thermal loads using an improved Halpin–Tsai micromechanical model, first-order shear deformation theory, and first-order piston theory. They obtained semi-analytic solutions for the critical flutter speed and critical buckling temperature by employing the differential quadrature method (DQM). Gao et al. [16] used the spectral geometry method to describe the displacement admissible functions of functionally graded graphene-reinforced composite conical shells, derived the dynamic equations of the conical shell structure based on the first-order shear deformation theory, and obtained the vibration characteristics by solving these dynamic equations. To address the vibration and buckling problems of truncated conical shells made of carbon nanotube-reinforced composites containing pores, Huang et al. [17] established the equations of motion for such truncated conical shells based on a modified model of the physical properties of porous composites, and solved them using the Galerkin integration method. Zhang et al. [18] analyzed the vibrational characteristics of thin-walled cylindrical shells reinforced with functionally graded graphene based on the first-order shear deformation theory, Halpin–Tsai micromechanical model, and Rayleigh–Ritz method. Zhang [19] investigated the stability and vibration characteristics of micrometer-scale cylindrical shells reinforced with graphene flakes based on the first-order shear deformation shell theory and strain gradient elasticity theory. Zhang et al. [20] used analytical methods to study the free vibration of an FG-GPLRC cylindrical shell, presented the analytical expressions, and quantitatively analyzed the effects of parameters such as the distribution pattern, content, and size of the graphene platelets, as well as the geometric parameters of the cylindrical shell, on natural frequencies. Wang et al. [21] established the dynamic equations for the rotating FG-GPLRC cylindrical shell based on the first-order shear deformation theory. Employing the Rayleigh–Ritz method, the traveling wave vibration behavior of the shells was examined, along with the effects of GPLs mass fraction, their five distribution patterns, layer number, and rotational speed on this behavior. Jian et al. [22] analyzed the vibration characteristics of functionally graded graphene-reinforced composite stepped cylindrical shells based on the Rayleigh–Ritz method. Based on first-order shear deformation theory, Sofiyev et al. [23] investigated the nonlinear vibration characteristics of an FG cylindrical shell resting on an elastic foundation. They assumed simply supported boundary conditions at the shell ends and adopted a single-mode vibration assumption, discretizing the shell’s displacement field into a trigonometric form. The impact of system parameters on the frequency response curves of cylindrical shells is evaluated via the numerical examples. Shao et al. [24] used the simplified first-order shear shell theory (S-FSDST) to study the vibration characteristics of laminated, ribbed, open-ended cylindrical shell structures. They obtained exact solutions using the method of reverberation ray matrix (MRRM). Gao et al. [25] combined Hamilton’s principle and the first-order shear deformation theory to derive the nonlinear partial differential governing equations of motion of the system. Li et al. [26] analyzed the natural frequencies of a damped cantilevered cylindrical shell based on the Flügge shell theory. Also, they presented the corresponding mode shape diagrams. Dong et al. [27] established a dynamic model of cylindrical shells based on Novozhilov’s shell theory and adopted a displacement field discretized by a combination of Chebyshev polynomials to investigate the traveling wave vibrational characteristics of spinning cylindrical shells under various boundary conditions. Similarly, Sharma [28] investigated the natural characteristics of fixed-free end cylindrical shells and validated the theoretical results against experiments, which showed good agreement.

Although extensive studies have separately investigated the mechanical behavior of graphene-reinforced shell structures, the dynamic response of porous structures, and the vibration of structures resting on elastic foundations, systematic research on visco-Pasternak foundations, complex boundary conditions, and the coupling effects of material and geometric parameters remains relatively scarce. To fill this research gap, this paper derives the governing vibration equations of graphene-reinforced porous cylindrical shells resting on a visco-Pasternak foundation based on the first-order shear deformation theory and Hamilton’s principle, and employs the differential quadrature method (DQM) to determine vibration frequencies under complex boundary conditions. Finally, the effects of boundary conditions, material parameters, geometric parameters, and foundation parameters on the vibration characteristics of porous cylindrical shells are systematically discussed.

The main innovations of this paper are as follows:

A visco-Pasternak foundation model is introduced, and the coupling effects of boundary conditions, material parameters, geometric parameters, and foundation parameters on the vibration characteristics of porous cylindrical shells are systematically investigated, revealing the interaction mechanisms among these factors.

The differential quadrature method (DQM) is employed to determine the vibration frequencies under complex boundary conditions.

## 2. Basic Equations

**Figure 1:**
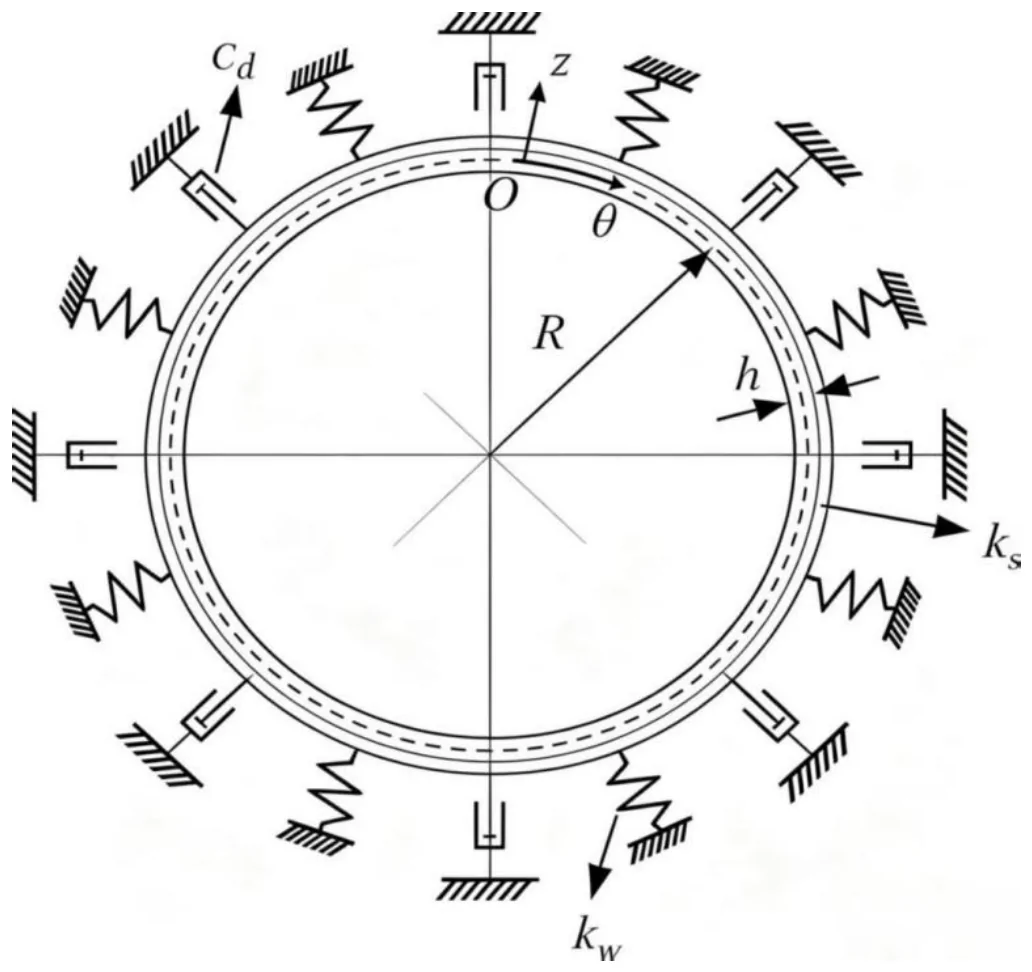
A graphene-reinforced porous cylindrical shell subjected to a visco-Pasternak foundation.

Resting on a visco-Pasternak foundation, Figure 1 illustrates a graphene-reinforced functionally graded porous cylindrical shell. A cylindrical coordinate system (*x*, *θ*, *z*) is introduced, with a point located at the mid-surface of the shell designated as the origin. In this system, the *x*-axis, *θ*-axis, and *z*-axis correspond to the axial, circumferential, and radial directions of the cylindrical shell, respectively. For the cylindrical shell, the length, radius, and thickness are represented by *L*, *R*, and *h*, while the elastic modulus, shear modulus, and damping coefficient are indicated as $k_{w}$, $k_{s}$, and $c_{d}$.

**Figure 2:**
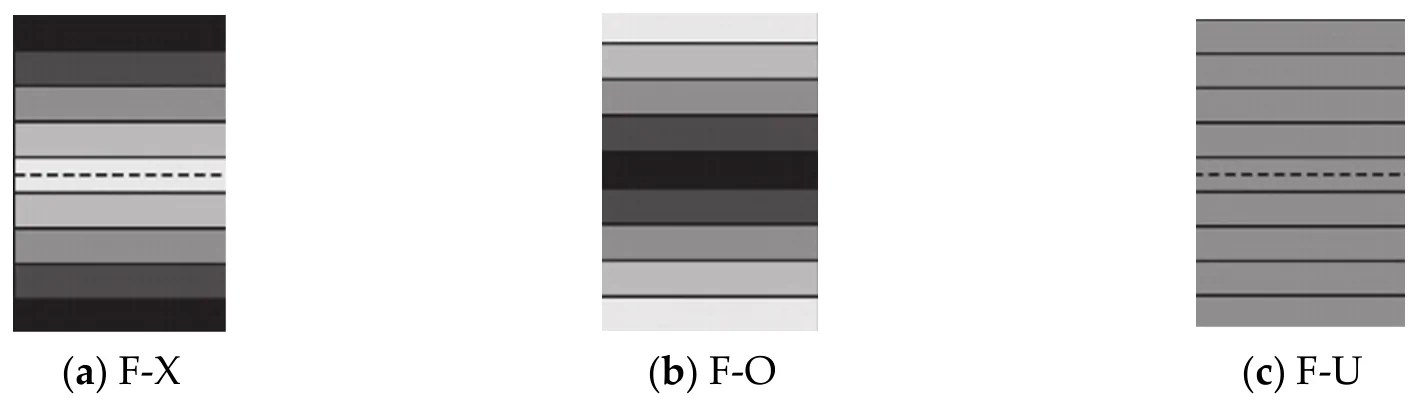
Graphene distribution patterns along the thickness direction.

This study investigates three graphene distribution configurations throughout the thickness of the cylindrical shell. As shown in Figure 2, the F-X mode features a higher concentration of graphene on the inner and outer surfaces of the cylindrical shell and a lower concentration in the middle; the F-O mode features a higher concentration of graphene in the middle of the cylindrical shell and a lower concentration on the inner and outer surfaces; and the F-U mode features a uniform distribution of graphene. The GPLs assumed to be perfectly bonded to the matrix and uniformly dispersed within each material layer.

For these three distribution models, the volume fraction of graphene $V_{G}$ is given by [29]

(1) $$V_{G}\left(z\right)=\left\{\begin{array}{c} V_{i1}\left(1-\cos(\pi z/h)\right)\;FG-X\\ V_{i2}\cos(\pi z/h)\;FG-O\\ V_{i3}\;FG-U \end{array}\right.$$

where $V_{i1}$, $V_{i2}$, and $V_{i3}$ are the volume fraction coefficients for the three graphene grading patterns under each pore distribution, with the subscripts *i* = 1, 2, and 3 corresponding to the three pore distributions, respectively.

The values of $V_{i1}$, $V_{i2}$, and $V_{i3}$ in Equation (1) can be derived using the following equation [29].

(2) $$V_{G}^{*}\int_{-h/2}^{h/2}\frac{\rho }{\rho_{0}}dz=\left\{\begin{array}{c} V_{i1}\int_{-h/2}^{h/2}\left[1-\cos\left(\frac{\pi z}{h}\right)\right]\frac{\rho }{\rho_{0}}dz\\ V_{i2}\int_{-h/2}^{h/2}\cos\left(\frac{\pi z}{h}\right)\frac{\rho }{\rho_{0}}dz\\ V_{i3}\int_{-h/2}^{h/2}\frac{\rho }{\rho_{0}}dz \end{array}\right.$$

In particular, the total volume fraction $V_{G}^{*}$ of graphene is defined as

(3) $$V_{G}^{*}=\frac{W_{G}}{W_{G}+\left(\rho_{G}/\rho_{M}\right)\left(1-W_{G}\right)}$$

where $\rho_{G}$ and $W_{G}$ are the density and mass fraction of graphene, respectively, and $\rho_{M}$ is the density of the matrix material.

According to the modified Halpin–Tsai micromechanical model [29], the elastic modulus $E_{0}$, mass density $\rho_{0}$ and Poisson’s ratio $\nu_{0}$ of a pore-free FG graphene-reinforced material can be expressed as

(4) $$E_{0}=(\frac{3}{8}\times \frac{1+\zeta_{L}\eta_{L}V_{G}}{1-\eta_{L}V_{G}}+\frac{5}{8}\times \frac{1+\zeta_{w}\eta_{w}V_{G}}{1-\eta_{w}V_{G}})E_{m}$$

(5) $$\rho_{0}=\rho_{G}V_{G}+\rho_{m}V_{m}$$

(6) $$\nu_{0}=\nu_{G}V_{G}+\nu_{m}V_{m}$$

where the variables with subscripts *G* and *m* represent physical parameters related to graphene and the matrix material, respectively.

The parameters, $\zeta_{L}$, $\zeta_{w}$, $\eta_{L}$, $\eta_{w}$ can be expressed as

(7) $$\zeta_{L}=\frac{2l_{G}}{h_{G}},\;\zeta_{w}=\frac{2w_{G}}{h_{G}},\;\eta_{L}=\frac{E_{G}-E_{m}}{E_{G}+\xi_{L}E_{m}},\;\eta_{w}=\frac{E_{G}-E_{m}}{E_{G}+\xi_{w}E_{m}}$$

where $l_{G}$, $w_{G}$, and $h_{G}$ are associated with the geometric dimensions of the graphene nanoplatelets, respectively, in meters (m).

**Figure 3:**
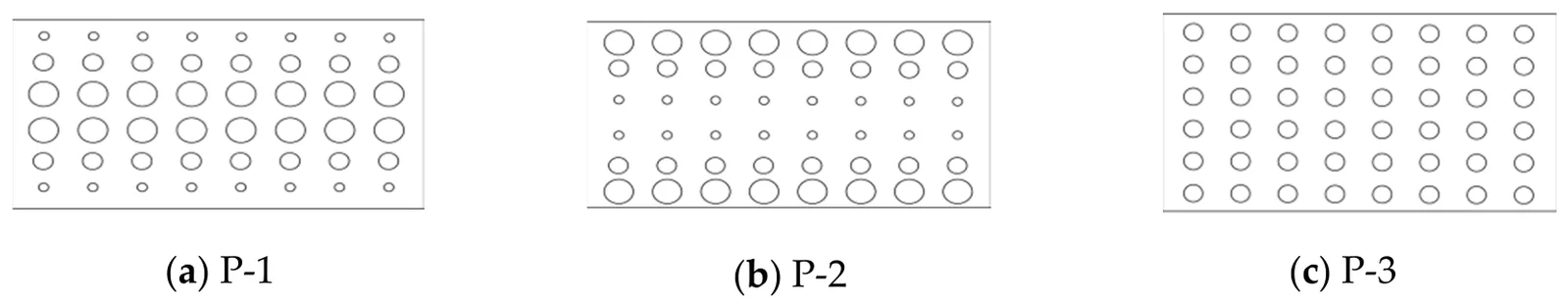
Different porosity distributions along the thickness direction.

The pores are assumed to be tiny and unconnected within the material, and the size effects are neglected. In addition, three porosity distributions along the cylindrical shell thickness are considered [6]: P-1, characterized by larger pores in the middle region and smaller pores near the inner and outer surfaces; P-2, exhibiting smaller pores in the middle region and larger pores near the inner and outer surfaces; and P-3, in which pores are uniformly distributed, as shown in Figure 3.

FG graphene-reinforced porous materials with three distributions of pores exhibit effective elastic modulus *E*, density *ρ*, and Poisson’s ratio *v*, which can be expressed as

P-1 Distribution

(8) $$\left\{\begin{array}{l} E=E_{0}\left[1-a_{1}\cos\left(\pi z/h\right)\right]\\ \rho =\rho_{0}\left[1-b_{1}\cos\left(\pi z/h\right)\right]\\ \nu =\nu_{0} \end{array}\right.$$

P-2 Distribution

(9) $$\left\{\begin{array}{l} E=E_{0}\left[1-a_{2}\left(1-\cos\left(\pi z/h\right)\right)\right]\\ \rho =\rho_{0}\left[1-b_{2}\left(1-\cos\left(\pi z/h\right)\right)\right]\\ \nu =\nu_{0} \end{array}\right.$$

P-3 Distribution

(10) $$\left\{\begin{array}{l} E=E_{0}a_{3}\\ \rho =\rho_{0}b_{3}\\ \nu =\nu_{0} \end{array}\right.$$

Specifically, $E_{0}$, $\rho_{0}$, and $\nu_{0}$ denote the intrinsic properties of the non-porous graphene-reinforced graded material; meanwhile, $a_{1}$ to $a_{3}$ and $b_{1}$ to $b_{3}$ signify the porosity and density factors corresponding to each pore distribution pattern, respectively.

The relationship between the modulus of elasticity and density is [30]

(11) $$\frac{E\left(z\right)}{E_{0}}={\left(\frac{\rho \left(z\right)}{\rho_{0}}\right)}^{2}$$

From Equation (11), the relationship between the porosity coefficients and the density coefficients for different pore distributions can be established, namely [31]

(12) $$\left\{\begin{array}{l} b_{1}=\left[1-\sqrt{1-a_{1}\cos\left(\pi z/h\right)}\right]/\cos\left(\pi z/h\right)\\ b_{2}=\left[1-\sqrt{1-a_{2}\left(1-\cos\left(\pi z/h\right)\right)}\right]/\left(1-\cos\left(\pi z/h\right)\right)\\ b_{3}=\sqrt{a_{3}} \end{array}\right.$$

To ensure comparability among different porosity distributions, the shell mass is assumed to be identical for all porosity distributions, yielding the following equation

(13) $$\left\{\begin{array}{l} \int_{0}^{h/2}\sqrt{1-a_{2}\left(1-\cos\left(\pi z/h\right)\right)}dz=\int_{0}^{h/2}\sqrt{1-a_{1}\cos\left(\pi z/h\right)}dz\\ \int_{0}^{h/2}\sqrt{a_{3}}dz=\int_{0}^{h/2}\sqrt{1-a_{1}\cos\left(\pi z/h\right)}dz \end{array}\right.$$

## 3. Governing Equations

Based on the first-order shear deformation theory [32], the displacement of any point on a cylindrical shell along the *x*, *θ*, and *z* directions can be expressed as

(14) $$\left\{\begin{array}{l} u(x,\theta ,z,t)=u_{0}(x,\theta ,t)+z\phi_{x}(x,\theta ,t)\\ v(x,\theta ,z,t)=v_{0}(x,\theta ,t)+z\phi_{\theta }(x,\theta ,t)\\ w(x,\theta ,z,t)=w_{0}(x,\theta ,t) \end{array}\right.$$

where the kinematic variables $u_{0}$, $\nu_{0}$, $w_{0}$, $\phi_{x}$, and $\phi_{\theta }$ define the mid-plane deflections and cross-sectional rotations, specifically about the *x*-axis and *θ*-axis, respectively. Time is denoted by *t*.

The strain components are defined by

(15) $$\left\{\begin{array}{l} \varepsilon_{xx}\\ \varepsilon_{\theta \theta }\\ \gamma_{x\theta }\\ \gamma_{xz}\\ \gamma_{\theta z} \end{array}\right\}=\left\{\begin{array}{l} \varepsilon_{xx}^{0}\\ \varepsilon_{\theta \theta }^{0}\\ \gamma_{x\theta }^{0}\\ \gamma_{xz}^{0}\\ \gamma_{\theta z}^{0} \end{array}\right\}+z\left\{\begin{array}{l} \kappa_{xx}\\ \kappa_{\theta \theta }\\ \kappa_{x\theta }\\ \kappa_{xz}\\ \kappa_{\theta z} \end{array}\right\}$$

where $\varepsilon_{xx}^{0}$, $\varepsilon_{\theta \theta }^{0}$, $\gamma_{x\theta }^{0}$, $\gamma_{xz}^{0}$, $\gamma_{\theta z}^{0}$ represent the mid-plane strain components, and $\kappa_{xx}$, $\kappa_{\theta \theta }$, $\kappa_{x\theta }$, $\kappa_{xz}$, $\kappa_{\theta z}$ represent the mid-plane curvatures, defined as follows

(16) $$\left\{\begin{array}{l} \varepsilon_{xx}^{0}=\frac{\partial u_{0}}{\partial x},\varepsilon_{\theta \theta }^{0}=(\frac{\partial v_{0}}{R\partial \theta }+\frac{w_{0}}{R})\\ \gamma_{x\theta }^{0}=(\frac{\partial u_{0}}{R\partial \theta }+\frac{\partial v_{0}}{\partial x}),\gamma_{xz}^{0}=(\frac{\partial w_{0}}{\partial x}+\phi_{x})\\ \gamma_{\theta z}^{0}=(\frac{\partial w_{0}}{R\partial \theta }-\frac{v_{0}}{R}+\phi_{\theta }),\kappa_{xx}=\frac{\partial \phi_{x}}{\partial x},\kappa_{\theta \theta }=\frac{\partial \phi_{\theta }}{\partial \theta }\\ \kappa_{x\theta }=(\frac{\partial \phi_{x}}{R\partial \theta }+\frac{\partial \phi_{\theta }}{\partial x}),\kappa_{xz}=0,\kappa_{\theta z}=0 \end{array}\right.$$

Based on the generalized Hooke’s law, the following simplified constitutive equation can be derived

(17) $$\left\{\begin{array}{l} \sigma_{xx}\\ \sigma_{\theta \theta }\\ \sigma_{\theta z}\\ \sigma_{xz}\\ \sigma_{x\theta } \end{array}\right\}=\left[\begin{array}{ccccc} Q_{11} & Q_{12} & 0 & 0 & 0\\ Q_{21} & Q_{22} & 0 & 0 & 0\\ 0 & 0 & Q_{44} & 0 & 0\\ 0 & 0 & 0 & Q_{55} & 0\\ 0 & 0 & 0 & 0 & Q_{66} \end{array}\right]\left\{\begin{array}{l} \varepsilon_{xx}\\ \varepsilon_{\theta \theta }\\ \gamma_{\theta z}\\ \gamma_{xz}\\ \gamma_{x\theta } \end{array}\right\}$$

in which $\sigma_{xx}$ and $\sigma_{\theta \theta }$ denote the normal stresses at any location within the shell along the *x* and *θ* directions, respectively, while $\sigma_{\theta z}$, $\sigma_{xz}$, and $\sigma_{x\theta }$ indicate the shear stresses acting on the *θz*, *xz*, and *xθ* planes. $Q_{ij}$ are the reduced stiffness coefficients that incorporate material parameters, and are expressed as

(18) $$\left\{\begin{array}{l} Q_{11}=Q_{22}=\frac{E}{1-\nu^{2}}\\ Q_{12}=Q_{21}=\frac{\nu E}{1-\nu^{2}}\\ Q_{44}=Q_{55}=Q_{66}=\frac{E}{2(1+\nu )} \end{array}\right.$$

In the *x*, *θ* directions, the internal forces, bending moments, and lateral shear forces perpendicular to the plane are provided by Equation (19).

(19) $$\left\{\begin{array}{l} \left\{N_{xx},N_{\theta \theta },N_{x\theta }\right\}=\int_{-h/2}^{h/2}\left\{\sigma_{xx},\sigma_{\theta \theta },\sigma_{x\theta }\right\}dz\\ \left\{M_{xx},M_{\theta \theta },M_{x\theta }\right\}=\int_{-h/2}^{h/2}\left\{\sigma_{xx},\sigma_{\theta \theta },\sigma_{x\theta }\right\}zdz\\ \left\{Q_{xz},Q_{\theta z}\right\}=\kappa \int_{-h/2}^{h/2}\left\{\sigma_{xz},\sigma_{\theta z}\right\}dz \end{array}\right.$$

where κ denotes the correction factor, which is set to 5/6 [15,33,34,35]. Further derivation yields

(20) $$\left(\begin{array}{l} N_{xx}\\ N_{\theta \theta }\\ N_{x\theta }\\ M_{xx}\\ M_{\theta \theta }\\ M_{x\theta }\\ Q_{\theta z}\\ Q_{xz} \end{array}\right)=\left(\begin{array}{cccccccc} A_{11} & A_{12} & 0 & B_{11} & B_{12} & 0 & 0 & 0\\ A_{12} & A_{22} & 0 & B_{12} & B_{22} & 0 & 0 & 0\\ 0 & 0 & A_{66} & 0 & 0 & B_{66} & 0 & 0\\ B_{11} & B_{12} & 0 & D_{11} & D_{12} & 0 & 0 & 0\\ B_{12} & B_{22} & 0 & D_{12} & D_{22} & 0 & 0 & 0\\ 0 & 0 & B_{66} & 0 & 0 & D_{66} & 0 & 0\\ 0 & 0 & 0 & 0 & 0 & 0 & \kappa A_{44} & 0\\ 0 & 0 & 0 & 0 & 0 & 0 & 0 & \kappa A_{55} \end{array}\right)\left(\begin{array}{l} \varepsilon_{xx}^{0}\\ \varepsilon_{\theta \theta }^{0}\\ \gamma_{x\theta }^{0}\\ \kappa_{xx}\\ \kappa_{\theta \theta }\\ \kappa_{x\theta }\\ \gamma_{\theta z}^{0}\\ \gamma_{xz}^{0} \end{array}\right)$$

where $A_{ij}$, $B_{ij}$, and $D_{ij}$ (i, j = 1, 2, 3, 4, 5, 6) represent the axial rigidity (owing to the symmetry of the material, $I_{1}$ = 0, $B_{ij}$ = 0), coupling rigidity, and flexural rigidity, respectively, and are expressed as

(21) $$(A_{ij},B_{ij},D_{ij})=\int_{-h/2}^{h/2}Q_{ij}(1,z,z^{2})dz$$

Hamilton’s principle serves as the foundation for deriving the governing equations of vibration.

(22) $$\int_{t1}^{t_{2}}\left(\delta U+\delta V-\delta K\right)dt=0$$

in which δU, δV, and δK denote the overall virtual strain energy, the total virtual work done by external loads, and the total virtual kinetic energy of the porous cylindrical shell, respectively.

The overall virtual strain energy is represented by

(23) $$\delta U=\int_{0}^{L}\int_{0}^{2\pi }\int_{-h/2}^{h/2}\left(\sigma_{xx}\delta \varepsilon_{xx}+\sigma_{\theta \theta }\delta \varepsilon_{\theta \theta }+\sigma_{x\theta }\delta \varepsilon_{x\theta }+k\sigma_{xz}\delta \varepsilon_{xz}+k\sigma_{\theta z}\delta \varepsilon_{\theta z}\right)Rdzd\theta dx$$

The total virtual effort exerted by external loads is represented by

(24) $$\delta V=\int_{0}^{L}\int_{0}^{2\pi }N_{Q}\delta w_{0}Rd\theta dx$$

where $N_{Q}$ denotes the load applied by the viscoelastic Pasternak medium to the circular cylinder, which is defined as

(25) $$N_{Q}=-k_{w}w_{0}+k_{s}\nabla^{2}w_{0}-c_{d}\frac{\partial w_{0}}{\partial t}$$

where $k_{w}$, $k_{s}$, and $c_{d}$ are the elastic modulus, shear modulus, and damping coefficient of the foundation, respectively; and $\nabla^{2}=\frac{\partial^{2}}{\partial x^{2}}+\frac{\partial^{2}}{\partial y^{2}}$ is the Laplace operator.

The total virtual kinetic energy is formulated as follows

(26) $$\delta K=\int_{0}^{L}\int_{0}^{2\pi }\int_{-h/2}^{h/2}\rho (z)\left(\dot{u}\delta \dot{u}+\dot{v}\delta \dot{v}+\dot{w}\delta \dot{w}\right)Rdzd\theta dx$$

Substituting the total virtual internal energy, total virtual work input, and total dynamic energy of the shell into Equation (22) yields the differential control equation of the structure, namely

(27) $$\left\{\begin{array}{l} \frac{\partial N_{xx}}{\partial x}+\frac{\partial N_{x\theta }}{R\partial \theta }=I_{0}{\ddot{u}}_{0}+I_{1}{\ddot{\phi }}_{x}\\ \frac{\partial N_{x\theta }}{\partial x}+\frac{\partial N_{\theta \theta }}{R\partial \theta }+\frac{Q_{\theta Z}}{R}=I_{0}{\ddot{v}}_{0}+I_{1}{\ddot{\phi }}_{\theta }\\ \frac{\partial Q_{xz}}{\partial x}+\frac{\partial Q_{\theta z}}{R\partial \theta }-\frac{N_{\theta \theta }}{R}+N_{Q}=I_{0}{\ddot{w}}_{0}\\ \frac{\partial M_{xx}}{\partial x}+\frac{\partial M_{x\theta }}{R\partial \theta }-Q_{xz}=I_{1}{\ddot{u}}_{0}+I_{2}{\ddot{\phi }}_{x}\\ \frac{\partial M_{x\theta }}{\partial x}+\frac{\partial M_{\theta \theta }}{R\partial \theta }-Q_{\theta z}=I_{1}{\ddot{v}}_{0}+I_{2}{\ddot{\phi }}_{\theta } \end{array}\right.$$

where the dot notation “¨” indicates the second derivative with respect to time, while $I_{i}$ represents the mass moments of inertia, given by

(28) $$I_{i}=\int_{-h/2}^{h/2}\rho (z)\cdot z^{i}dz\;(i=0,1,2)$$

## 4. Solving Process

To facilitate the subsequent discussion, the following symbols are employed to designate the various boundary conditions: C denotes clamped, S signifies simply supported, and F indicates free.

### 4.1. Exact Solution

For a structure with simply supported ends, the boundary constraints are given by

(29) $$N_{x\left(x=0,L\right)}=v_{0\left(x=0,L\right)}=w_{0\left(x=0,L\right)}=M_{x\left(x=0,L\right)}=\phi_{\theta \left(x=0,L\right)}=0$$

To fulfill the S-S constraints, the displacement function reads

(30) $$\left\{\begin{array}{l} u_{0}(x,\theta ,t)=U_{mn}\cos\frac{m\pi x}{L}\sin(n\theta )e^{i\omega t}\\ v_{0}(x,\theta ,t)=V_{mn}\sin\frac{m\pi x}{L}con(n\theta )e^{i\omega t}\\ w_{0}(x,\theta ,t)=W_{mn}\sin\frac{m\pi x}{L}\sin(n\theta )e^{i\omega t}\\ \phi_{x}(x,\theta ,t)=\varphi_{xmn}\cos\frac{m\pi x}{L}\sin(n\theta )e^{i\omega t}\\ \phi_{y}(x,\theta ,t)=\varphi_{ymn}\sin\frac{m\pi x}{L}\cos(n\theta )e^{i\omega t} \end{array}\right.$$

where *m* denotes the half-range wavenumber in the axial direction, while *n* signifies the wavenumber along the circumference. $U_{mn}$, $V_{mn}$, $W_{mn}$, $\varphi_{xmn}$, $\varphi_{ymn}$ are the vibration amplitudes, ω is the angular frequency, and *t* is time.

Substituting Equation (30) into Equation (27) yields a homogeneous linear system of equations in terms of the amplitudes.

(31) $$\left(K-\omega^{2}M+\omega C\right)X=0$$

where X=${\left[U_{mn},V_{mn},W_{mn},\varphi_{xmn},\varphi_{ymn}\right]}^{T}$, *K*, *M*, and *C* denote the stiffness matrix, mass matrix, and damping matrix, respectively; the damping coefficient (which gives rise to the imaginary part) is contained in the damping matrix. For the linear system to have a nonzero solution, the determinant of its coefficient matrix must be zero, from which the characteristic equation is obtained. The characteristic equation was solved using MATLAB (2021a), and the eigenvalue *ω* is $e^{i\omega t}=e^{i(a+bi)t}$, where the real part a is the natural frequency and the imaginary part b is the damping factor. In this paper, only the natural frequency corresponding to the real part is considered. Then, the angular frequency *ω* is converted to f (Hz), that is, f=ω/2π.

### 4.2. Numerical Solution

Analytical solutions for complicated boundary constraints such as C-C, C-S, and C-F are often intractable, necessitating the use of numerical approaches. In this study, the differential quadrature method (DQM) is employed to obtain the numerical solution, due to the following reasons: First, it enables high-order derivative estimates even with a coarse discretization. balancing computational efficiency and numerical accuracy. Second, its discrete formulation is well-suited for high-order partial differential equations, matching the inherent nature of the vibrational governing equations.

The boundary conditions are formulated as follows (the detailed treatment is illustrated by taking Equation (34) as an example; see Appendix A)

(32) $$\left\{\begin{array}{l} S:N=v_{0}=w_{0}=M_{x}=\phi_{\theta }=0\\ C:u_{0}=v_{0}=w_{0}=\phi_{x}=\phi_{\theta }=0\\ F:N_{x}=N_{x\theta }=Q_{x}=M_{x}=M_{x\theta }=0 \end{array}\right.$$

Assume the displacement functions under complex boundary conditions as

(33) $$\left\{\begin{array}{l} u_{0}\left(x,\theta ,t\right)=U\left(x\right)\sin\left(n\theta \right)e^{i\omega t}\\ v_{0}\left(x,\theta ,t\right)=V\left(x\right)\cos\left(n\theta \right)e^{i\omega t}\\ w_{0}\left(x,\theta ,t\right)=W\left(x\right)\sin\left(n\theta \right)e^{i\omega t}\\ \phi_{x}\left(x,\theta ,t\right)=X\left(x\right)\sin\left(n\theta \right)e^{i\omega t}\\ \phi_{y}\left(x,\theta ,t\right)=Y\left(x\right)\cos\left(n\theta \right)e^{i\omega t} \end{array}\right.$$

In the above equations, the axial mode shapes correspond to *U*(*x*), *V*(*x*), *W*(*x*), *X*(*x*), and *Y*(*x*) for the five deformation directions, and *n* is the circumferential wavenumber.

Combining Equation (33) with Equation (27) results in a system of governing partial differential equations involving the displacement functions.

(34) $$\begin{array}{l} A_{11}\frac{\partial U^{2}(x)}{\partial x^{2}}-[\frac{n^{2}A_{66}}{R^{2}}-I_{0}\omega^{2}]U(x)-\frac{n}{R}[A_{12}+A_{66}]\frac{\partial V(x)}{\partial x}+\frac{A_{12}}{R}\frac{\partial W(x)}{\partial x}+\\ B_{11}\frac{\partial X^{2}(x)}{\partial x^{2}}-[\frac{n^{2}B_{66}}{R^{2}}-I_{1}\omega^{2}]X(x)-\frac{n}{R}[B_{12}+B_{66}]\frac{\partial Y(x)}{\partial x}=0 \end{array}$$

(35) $$\begin{array}{l} \frac{n}{R}[A_{12}+A_{66}]\frac{\partial U(x)}{\partial x}+A_{66}\frac{\partial V^{2}(x)}{\partial x^{2}}-[\frac{n^{2}A_{22}}{R^{2}}+k\frac{A_{44}}{R^{2}}-I_{0}\omega^{2}]V(x)+[\frac{nA_{22}}{R^{2}}+\frac{knA_{44}}{R^{2}}]W(x)+\\ \frac{n}{R}[B_{12}+B_{66}]\frac{\partial X(x)}{\partial x}+B_{66}\frac{\partial Y^{2}(x)}{\partial x^{2}}-[\frac{n^{2}B_{22}}{R^{2}}-k\frac{A_{44}}{R}-I_{1}\omega^{2}]Y(x)=0 \end{array}$$

(36) $$\begin{array}{l} -\frac{A_{12}}{R}\frac{\partial U(x)}{\partial x}+\frac{n}{R^{2}}[A_{22}+kA_{44}]V(x)+[kA_{55}+k_{s}]\frac{\partial W^{2}(x)}{\partial x^{2}}-\\ {}[\frac{n^{2}kA_{44}}{R^{2}}+\frac{A_{22}}{R^{2}}-I_{0}\omega^{2}+k_{w}+\frac{k_{s}n^{2}}{R^{2}}+c_{d}\omega i]W(x)+[kA_{55}-\frac{B_{12}}{R}]\frac{\partial X(x)}{\partial x}-\frac{n}{R}[kA_{44}-\frac{B_{22}}{R}]Y(x)=0 \end{array}$$

(37) $$\begin{array}{l} B_{11}\frac{\partial U^{2}(x)}{x^{2}}-[\frac{n^{2}B_{66}}{R^{2}}-I_{1}\omega^{2}]U(x)-\frac{n}{R}[B_{12}+B_{66}]\frac{\partial V(x)}{\partial x}+[\frac{B_{12}}{R}-kA_{55}]\frac{\partial W(x)}{\partial x}+\\ D_{11}\frac{\partial X^{2}(x)}{\partial x^{2}}-[\frac{n^{2}D_{66}}{R^{2}}+kA_{55}-I_{2}\omega^{2}]X(x)-\frac{n}{R}[D_{12}+D_{66}]\frac{\partial Y(x)}{\partial x}=0 \end{array}$$

(38) $$\begin{array}{l} \frac{n}{R}[B_{12}+B_{66}]\frac{\partial U(x)}{\partial x}+B_{66}\frac{\partial V^{2}(x)}{\partial x^{2}}-[\frac{n^{2}B_{22}}{R^{2}}-\frac{kA_{44}}{R}-I_{1}\omega^{2}]V(x)+\frac{n}{R}[\frac{B_{22}}{R}-kA_{44}]W(x)+\\ \frac{n}{R}[D_{12}+D_{66}]\frac{\partial X(x)}{\partial x}+D_{66}\frac{\partial Y^{2}(x)}{\partial x^{2}}-[\frac{n^{2}D_{22}}{R^{2}}+kA_{44}-I_{2}\omega^{2}]Y(x)=0 \end{array}$$

By utilizing the differential quadrature method [36], the derivatives with respect to *x* are expressed as weighted sums of the function values at discrete points.

(39) $$\frac{d^{r}}{dx^{r}}\left\{\begin{array}{c} U(x_{i})\\ V(x_{i})\\ W(x_{i})\\ X(x_{i})\\ Y(x_{i}) \end{array}\right\}=\sum_{j=1}^{N}C_{ij}^{r}\left\{\begin{array}{c} U(x_{j})\\ V(x_{j})\\ W(x_{j})\\ X(x_{j})\\ Y(x_{j}) \end{array}\right\}$$

where the weighting coefficient $C_{ij}^{r}$ can be calculated using the following equation

(40) $$\left\{\begin{array}{l} C_{ij}^{\left(1\right)}=\frac{\prod_{j=1,j\neq i}^{N}\left(x_{i}-x_{j}\right)}{(x_{i}-x_{j})\prod_{k=1,k\neq i}^{N}\left(x_{j}-x_{k}\right)},(i,j=1,2,\ldots \ldots ,N)\\ C_{ij}^{\left(r\right)}=r\left[-C_{ij}^{\left(1\right)}\sum_{j=1,j\neq i}^{N}C_{ij}^{r-1}-\frac{C_{ij}^{r-1}}{x_{i}-x_{j}}\right],(r=2,3,\ldots \ldots \ldots ,N-1) \end{array}\right.$$

where *r* denotes the order of the derivative, $U\left(x_{j}\right)$, $V\left(x_{j}\right)$, $W\left(x_{j}\right)$, $X\left(x_{j}\right)$, and $Y\left(x_{j}\right)$ denote the function values at each node $x_{j}$ after discretization, and *N* represents the total count of discrete points in the *x*-direction.

To ensure the accuracy of the calculation results, we choose non-uniform Chebyshev discrete points.

(41) $$\xi_{i}=\frac{L\left\{1-\cos\left[\frac{(i-1)\pi }{N-1}\right]\right\}}{2},(i=1,2,\ldots \ldots \ldots N)$$

Applying the DQM to the system of high-order partial differential equations, we can rewrite it as

(42) $$\left(K-\omega^{2}M+\omega C\right)\delta =0$$

where *M* is the mass matrix, *K* is the stiffness matrix, *C* is the damping matrix, and δ is the nodal displacement vector. By solving the characteristic equation $\left|K-\omega^{2}M+\omega C\right|=0$ through MATLAB programming, the natural frequencies of the numerical solution can be obtained.

## 5. Verification

In this section, we first simplify the model and benchmark the obtained results against existing studies to validate the proposed theory. If the effect of the visco-Pasternak foundation is neglected ($k_{w}=k_{s}=c_{d}=0$), the developed model degenerates into the classical FG graphene-reinforced porous cylindrical shell model.

### 5.1. Verification of the Exact Solution

This example examines the free vibration behavior of a cylindrical shell featuring simply supported ends. The matrix material is steel, while graphene serves as the reinforcement. The properties of the materials are listed in Table 1. The graphene content is set to 1%, the geometric parameters of the graphene nanoplatelets are $l_{G}=2.5\times 10^{-6}$ m, $w_{G}=1.5\times 10^{-6}$ m, $h_{G}=1.5\times 10^{-9}$ m, and the graphene gradient distribution pattern is F-X. The pore distribution is P-1, and the porosity coefficient $a_{1}$ = 0.5. The geometric parameters of the porous cylindrical shell are *R* = 1 m, *L* = 20 m, and *h* = 0.002 m.

The exact solutions for the natural frequencies calculated in this paper are compared with the results in Refs. [31,37], presented in Table 2.

As shown in Table 2, the natural frequencies calculated in this paper differ from those reported in Refs. [31,37] by a maximum of 5.6% and 0.23%, respectively, indicating good agreement with available results. Therefore, the theoretical model and programming presented in this paper are correct.

### 5.2. Verification of Numerical Solutions

**Figure 4:**
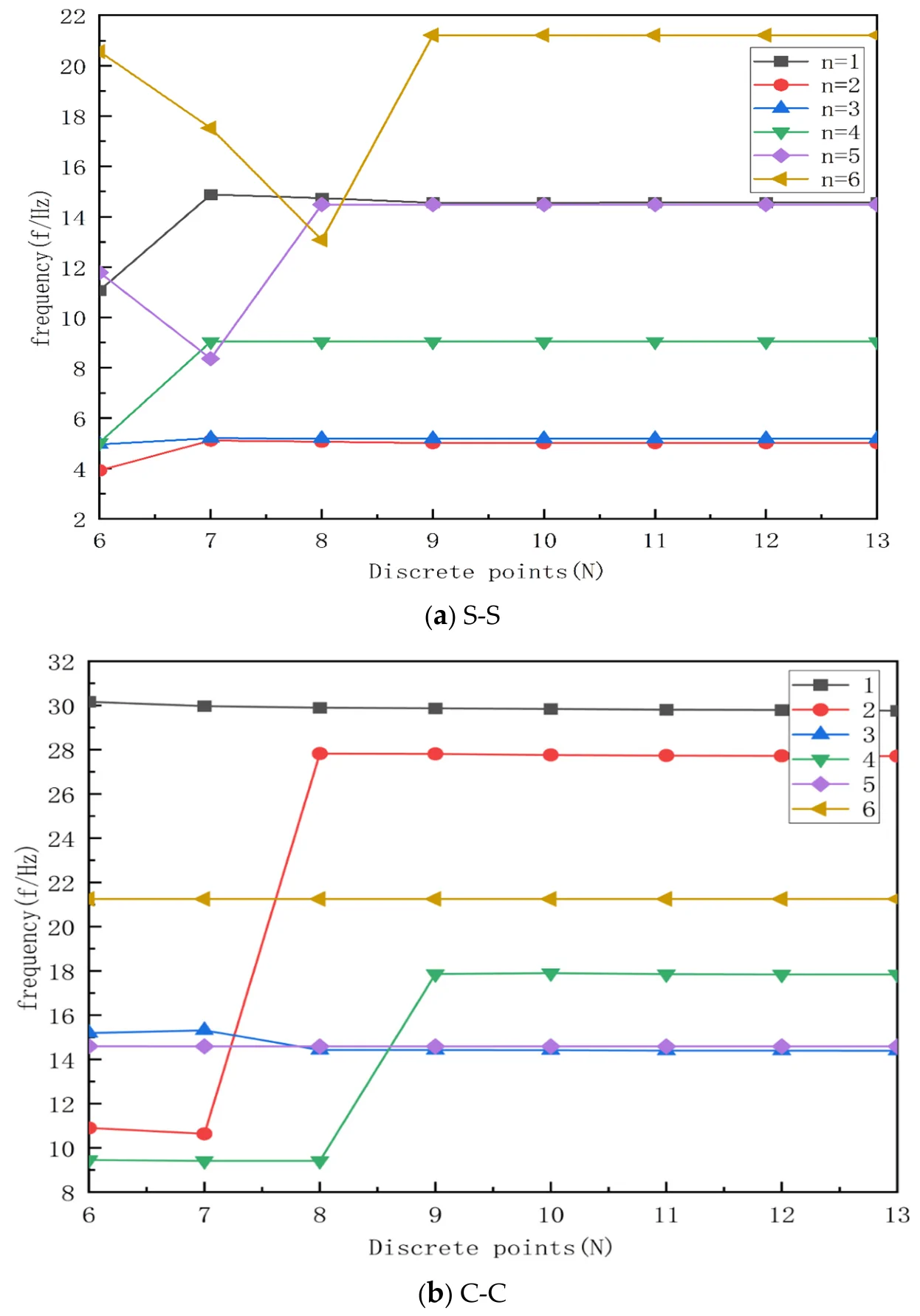
Convergence analysis.

We begin by examining how the natural frequencies evolve as the number of discrete points *N* varies, considering a cylindrical shell with simply supported ends. The material and geometric parameters are the same with Section 4.1. Figure 4 illustrates that the frequency stabilizes once *N* reaches 9. Consequently, to maintain precision, a value of *N* = 12 is adopted for all subsequent analyses.

To verify whether the computational results obtained using the differential quadrature method are accurate and reliable, we have computed the frequencies for *N* = 12 discrete points, axial wavenumber *m* = 1, and circumferential wavenumbers *n* = 3, 4, and 5, and compared them with the exact solutions mentioned above, as shown in Table 3.

As seen from Table 3, the numerical solutions obtained by DQM are very close to the exact solutions. This verifies the accuracy of the differential quadrature method, which can thus be employed for semi-analytical solutions hereafter.

To verify the convergence behavior under complex boundary conditions, a clamped-clamped case is adopted for convergence validation, with the results presented in Figure 4b. It can be observed that its convergence trend is consistent with the simply supported boundary: when the number of discrete points reaches *N* = 9, the calculated natural frequencies of the cylindrical shell tend to constant values.

## 6. Examples and Discussion

Upon confirming the validity of the proposed formulation and completing the programming, this section investigates the dynamic responses of the cylindrical shell. The matrix is steel, and the reinforcement is graphene, with the material parameters given in Table 1. Unless otherwise specified, the geometric dimensions of the graphene nanoplatelets are $l_{G}=2.5\times 10^{-6}$ m, $w_{G}=1.5\times 10^{-6}$ m and $h_{G}=1.5\times 10^{-9}$ m. The material has a porosity coefficient of *a* = 0.3, a graphene content of 1%, a graphene distribution pattern of F-X, and a pore distribution pattern of P-3. The geometric dimensions of a porous cylindrical shell are *R* = 1 m, *h* = 0.01 m, and *L* = 20 m. Since no nondimensionalization was performed, these numerical results are valid only within the given computational framework.

### 6.1. Effect of Boundary Conditions

**Figure 5:**
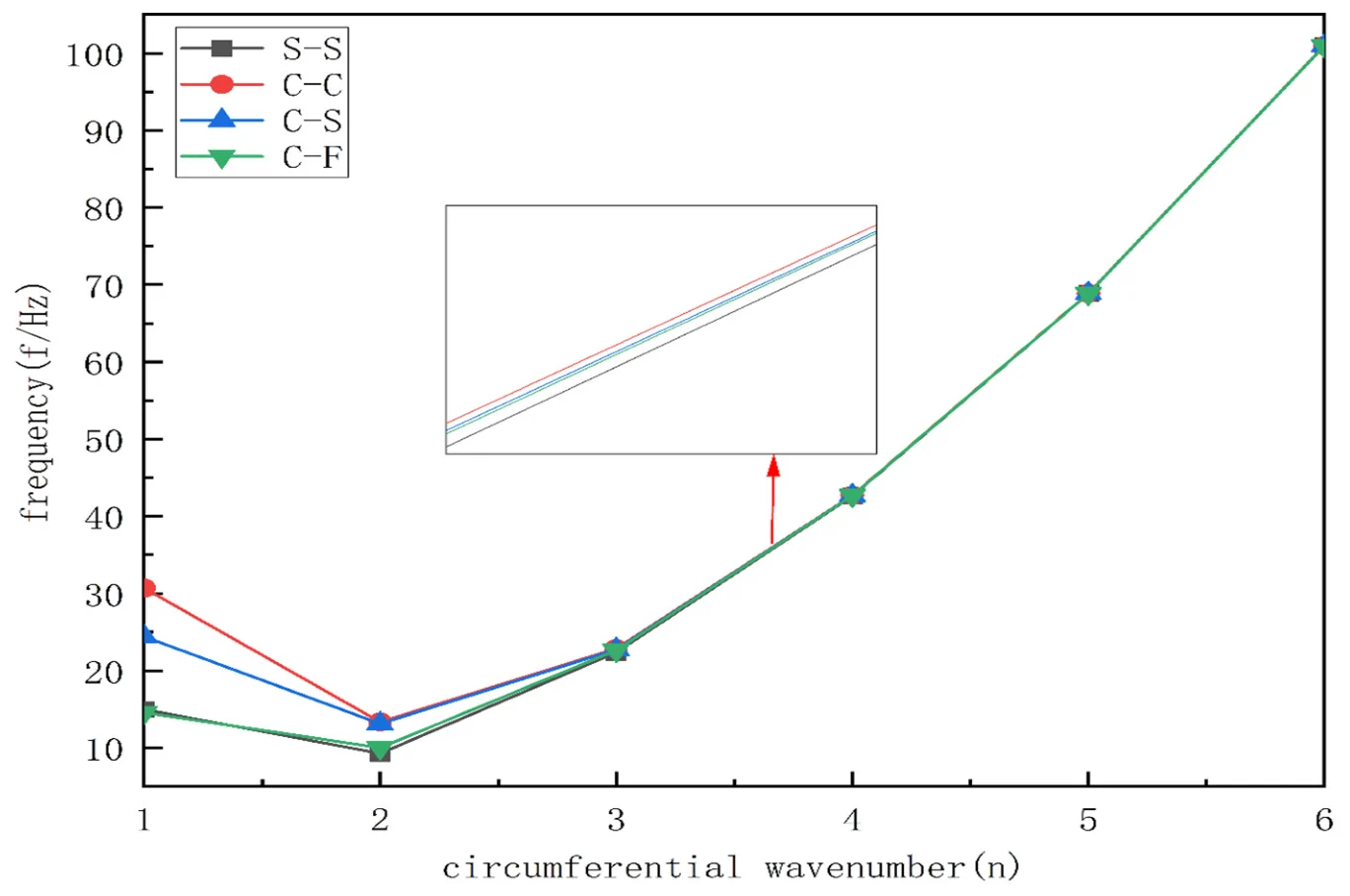
Frequency variation versus circumferential wavenumber for various boundary conditions.

The variation in the vibration frequencies of cylindrical shells with the circumferential wavenumber *n* under different boundary conditions is shown in Figure 5. It can be seen from the figure that, under the four boundary conditions, when *m* = 1 and *n* ranges from 1 to 6, the vibration frequencies all exhibit a trend of first decreasing and then increasing with the increase in the circumferential wavenumber *n*; in this series of modes, the lowest frequency occurs at (*m* = 1, *n* = 2) rather than at (*m* = 1, *n* = 1). The frequency differences among different boundary conditions decrease with the increase in the circumferential wavenumber *n*, and the larger the circumferential wavenumber, the smaller the differences, indicating that the influence of boundary conditions on the vibration frequency gradually weakens with the increase in the circumferential wavenumber. From the enlarged view in Figure 5, it can be seen that the vibration frequency under the C-C boundary condition always remains the highest value.

### 6.2. Effect of Material Parameters

Table 4 illustrates how the vibration frequencies of cylindrical shells are influenced by both the graphene distribution pattern and its content across various boundary conditions. It is apparent that the boundary constraints dictate the frequency values, with the clamped-clamped (C-C) case producing the maximum response. Among the three graphene distribution patterns, F-X exhibits the greatest structural stiffness and the highest vibration frequency, indicating that the F-X distribution provides a more effective enhancement of material stiffness than F-O and F-U. Meanwhile, an augmentation in graphene content corresponds to a rise in the cylindrical shell’s vibration frequency because graphene has extremely high stiffness and strength. The higher its content in the matrix material, the greater the overall stiffness of the material.

**Figure 6:**
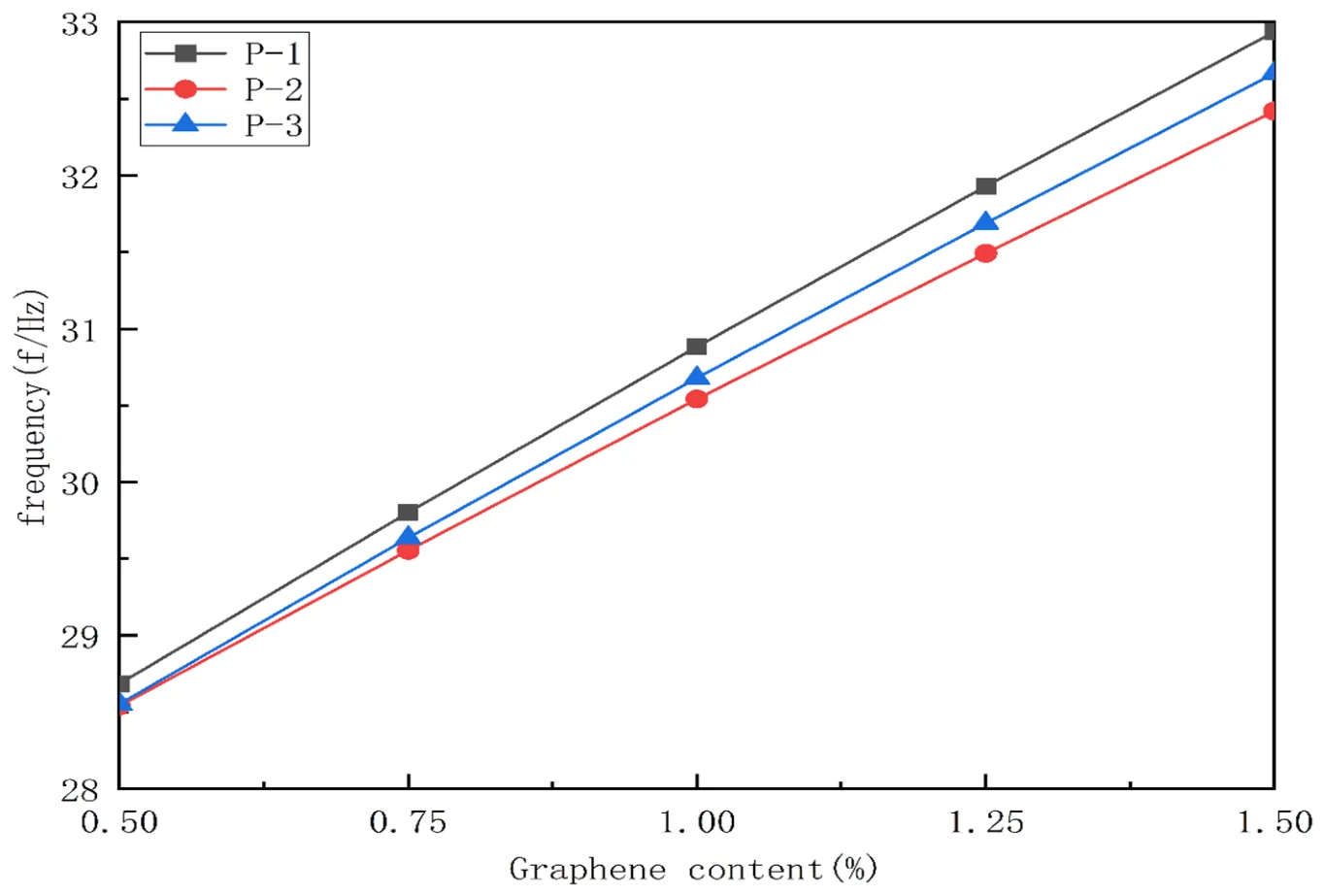
Frequency versus graphene content for three porosity distributions.

Figure 6 depicts how the vibration frequency of the cylindrical shell changes alongside graphene content across various porosity distributions under the C-C boundary condition. The data indicate a consistent upward trend, with similar slopes observed for different distributions. The porous cylindrical shell with the P-1 distribution exhibits the highest vibration frequency and thus possesses the greatest structural stiffness, followed by the P-3 and P-2 distributions; the sequence is not affected by the graphene content. The discrepancies in vibration frequencies among cylindrical shells with different porosity distributions are primarily attributed to their distinct effects on the effective stiffness. Among these, the P-1 distribution exhibits the weakest degrading effect. In engineering applications, P-1 cylindrical shells with low porosity at the upper and lower surfaces and high porosity in the middle are more suitable for use as structural components.

**Figure 7:**
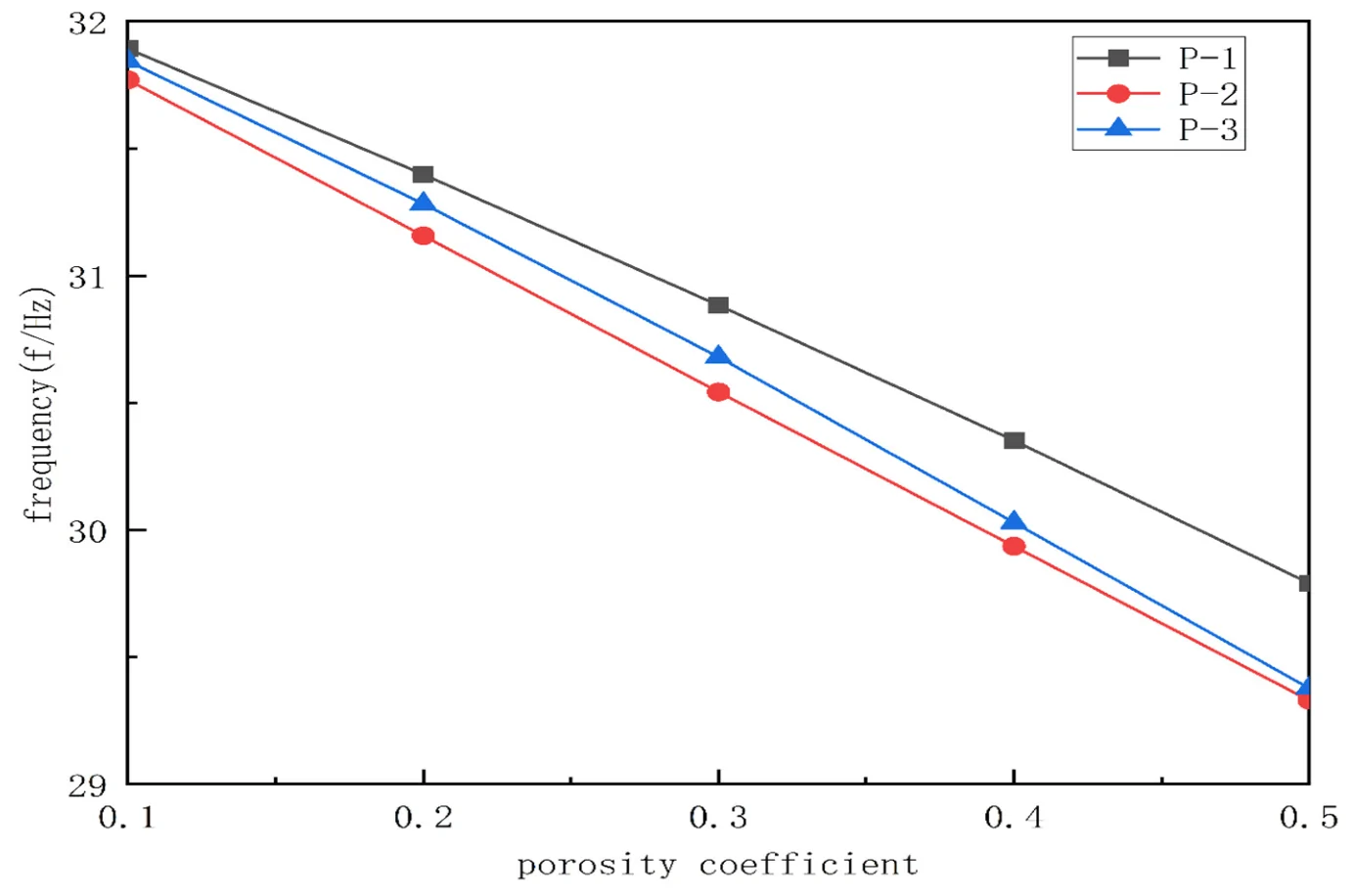
Frequency versus porosity coefficient for three porosity distributions.

Figure 7 illustrates how the vibration frequency of the cylindrical shell responds to changes in the porosity coefficient across various porosity distributions under the C-C boundary condition. Figure 7 clearly reveals a general trend in which the vibration frequencies of all three porosity distributions exhibit a decreasing tendency as the porosity coefficient increases. Among them, P-3 is the most sensitive to changes in the porosity coefficient (with the largest absolute slope), followed by P-2, while P-1 shows the relatively lowest sensitivity. As the porosity coefficient rises, the structural stiffness diminishes, thereby causing a decrease in the vibration frequency. This effect is stronger than that of the porosity distribution; that is, in engineering applications, adjusting the porosity coefficient of the material can be prioritized to alter the structural stiffness.

**Figure 8:**
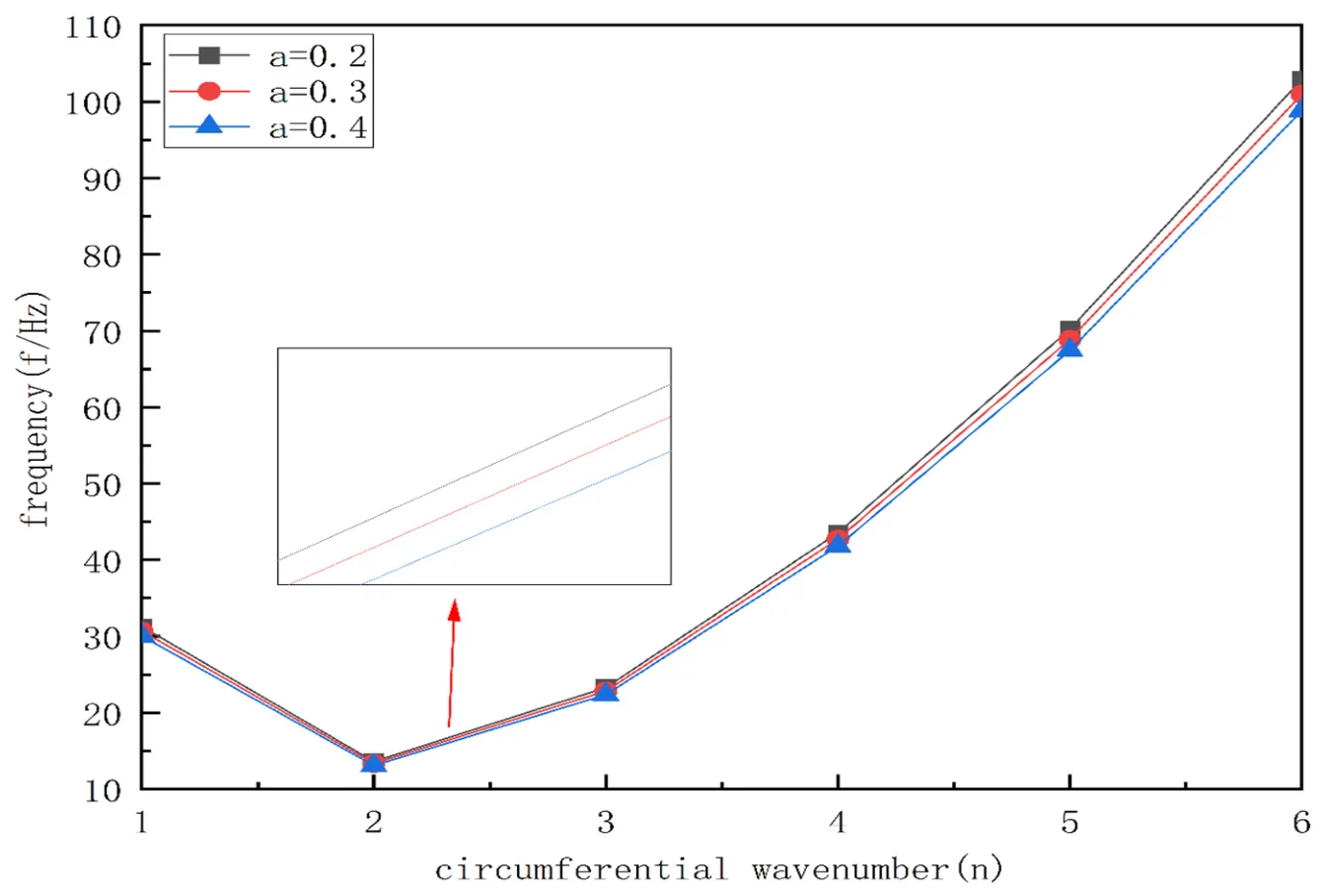
Frequency versus circumferential wavenumber for different porosity coefficients.

Figure 8 presents the fluctuation of vibration frequency relative to the circumferential wavenumber *n* for cylindrical shells exhibiting different porosity coefficients subject to C-C boundary conditions. Regardless of the coefficient value, the frequency initially drops before rising again as *n* progresses. A closer look at the embedded graph reveals a declining trend in frequency alongside an increasing porosity coefficient. This behavior can be attributed to the fact that a higher porosity coefficient signifies a greater number of pores, thereby compromising the structural stiffness. Furthermore, the impact of the porosity coefficient becomes more evident with ascending circumferential wavenumber *n*.

**Figure 9:**
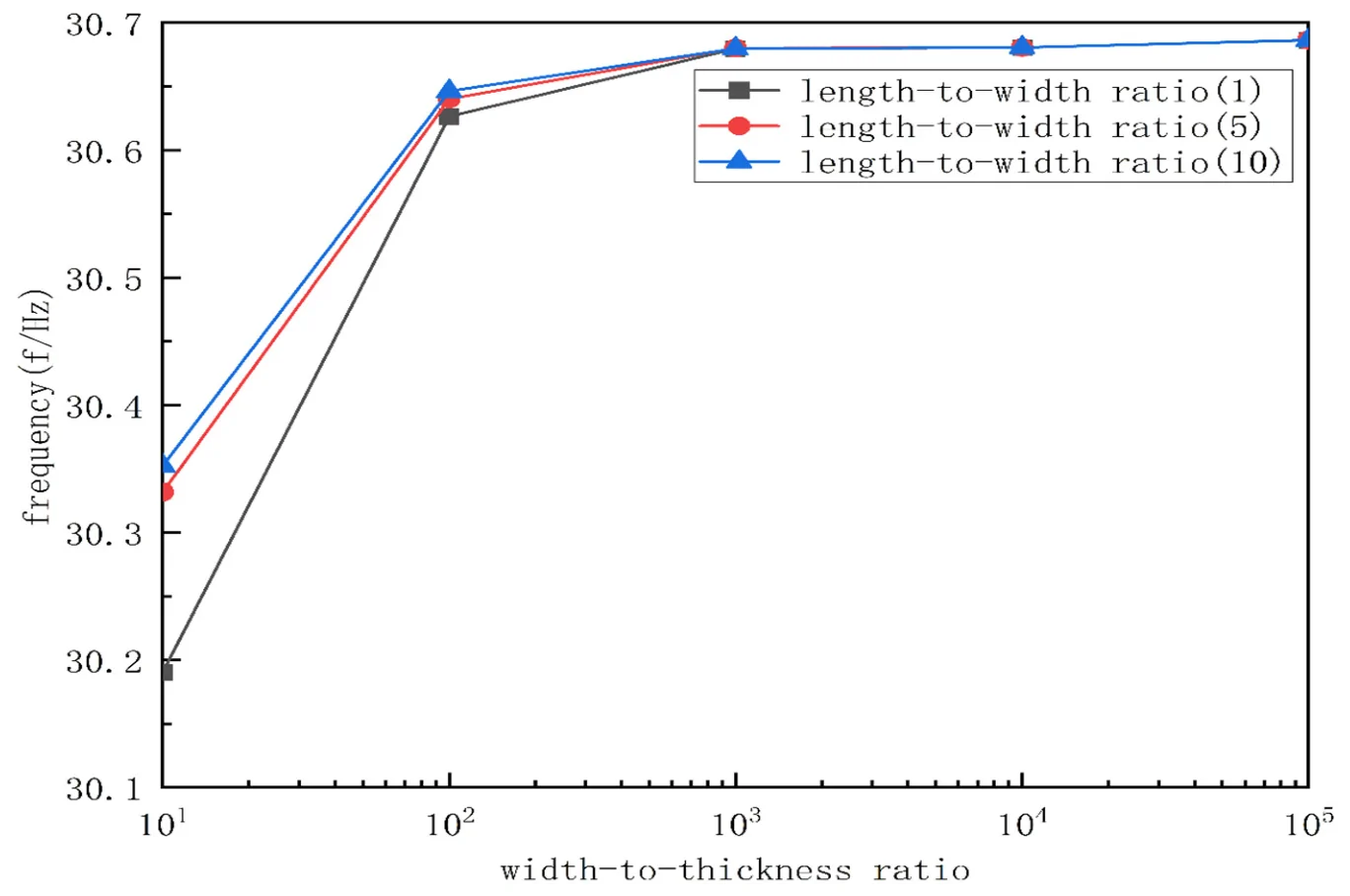
Effect of geometric dimensions of GPLs on natural frequencies.

Figure 9 investigates how the geometric dimensions of graphene nanoplatelets affect the vibration frequency of cylindrical shells subject to C-C boundary conditions. As depicted in Figure 9, the vibration frequency rises with the elevated width-to-thickness ratio and aspect ratio of the graphene nanoplatelets. This is due to the larger interfacial surface between the matrix and graphene nanoplatelets, which improves load transfer, resulting in higher stiffness and thus higher vibration frequencies. Beyond a width-to-thickness ratio of 1000, the vibration frequency plateaus, while the impact of both the width-to-thickness ratio and the aspect ratio diminishes. Conversely, below this threshold, a reduced aspect ratio correlates with a more pronounced influence of the width-to-thickness ratio on the vibration frequency. In contrast, elevating the aspect ratio attenuates this effect.

### 6.3. Effect of Geometric Parameters

**Figure 10:**
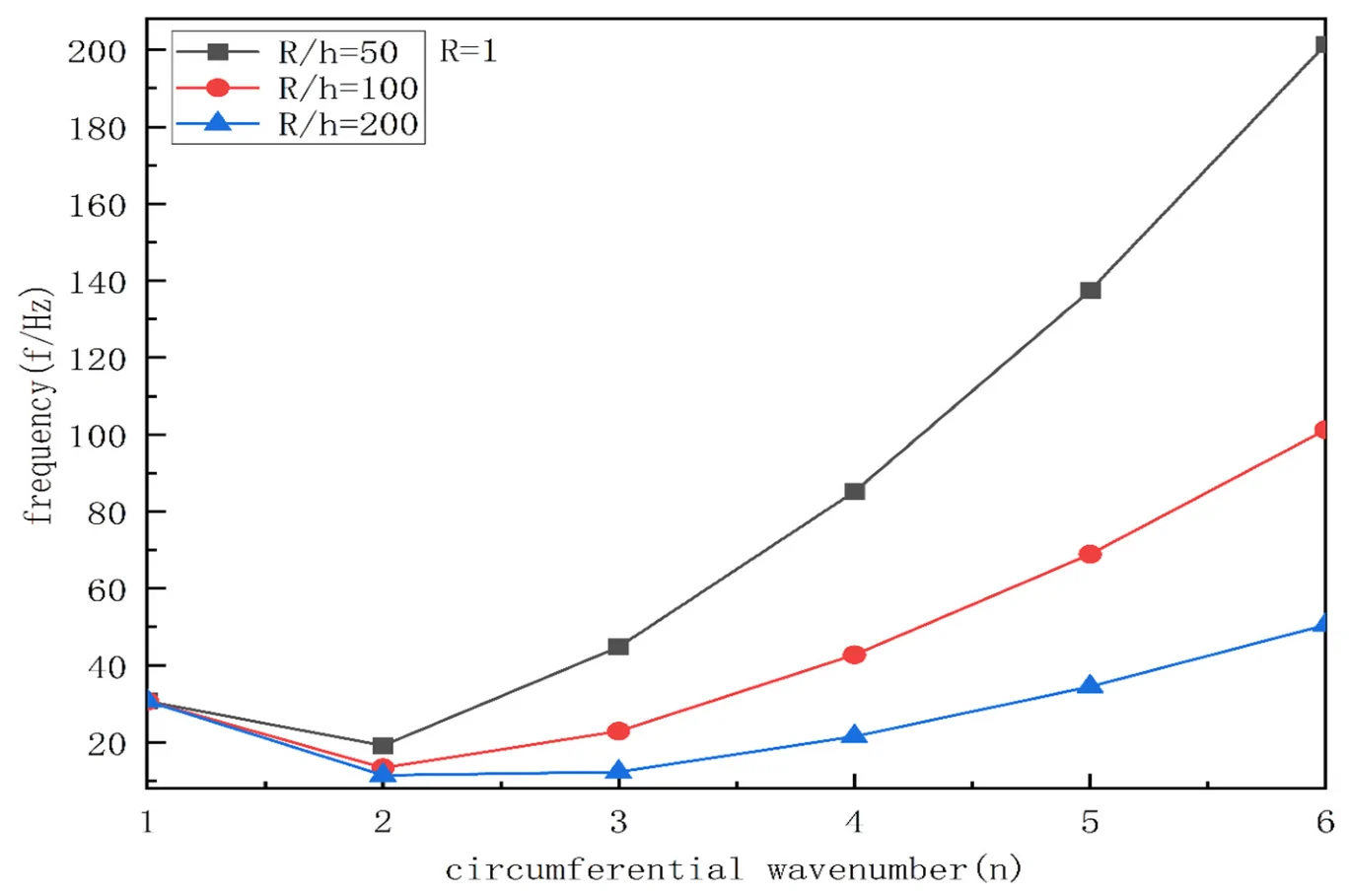
Frequency versus circumferential wavenumber for different radius-to-thickness ratios.

For a given boundary condition C-C, varying the shell thickness h, the corresponding vibration frequencies for different circumferential wavenumbers *n* are shown in Figure 10. It can be seen from the figure that the vibration frequency decreases with increasing radius-to-thickness ratio. When *n* = 2, the frequency decreases with increasing circumferential wavenumber, and the variation amplitude is relatively small; the sensitivity of the vibration frequency to changes in the radius-to-thickness ratio is low. When *n* = 3, the vibration frequency increases with increasing circumferential wavenumber, and the variation amplitude is large; the sensitivity of the vibration frequency to changes in the radius-to-thickness ratio is high. In particular, at the fundamental frequency (*m* = 1, *n* = 1), the vibration frequency is almost unaffected by the radius-to-thickness ratio. This may be because the strain energy of this mode is dominated by membrane deformation, and both the membrane stiffness and mass vary linearly with thickness, making the frequency insensitive to thickness changes. The smaller the radius-to-thickness ratio, the larger the frequency differences between modes of different orders, and the more pronounced the order differentiation of structural vibrations.

**Figure 11:**
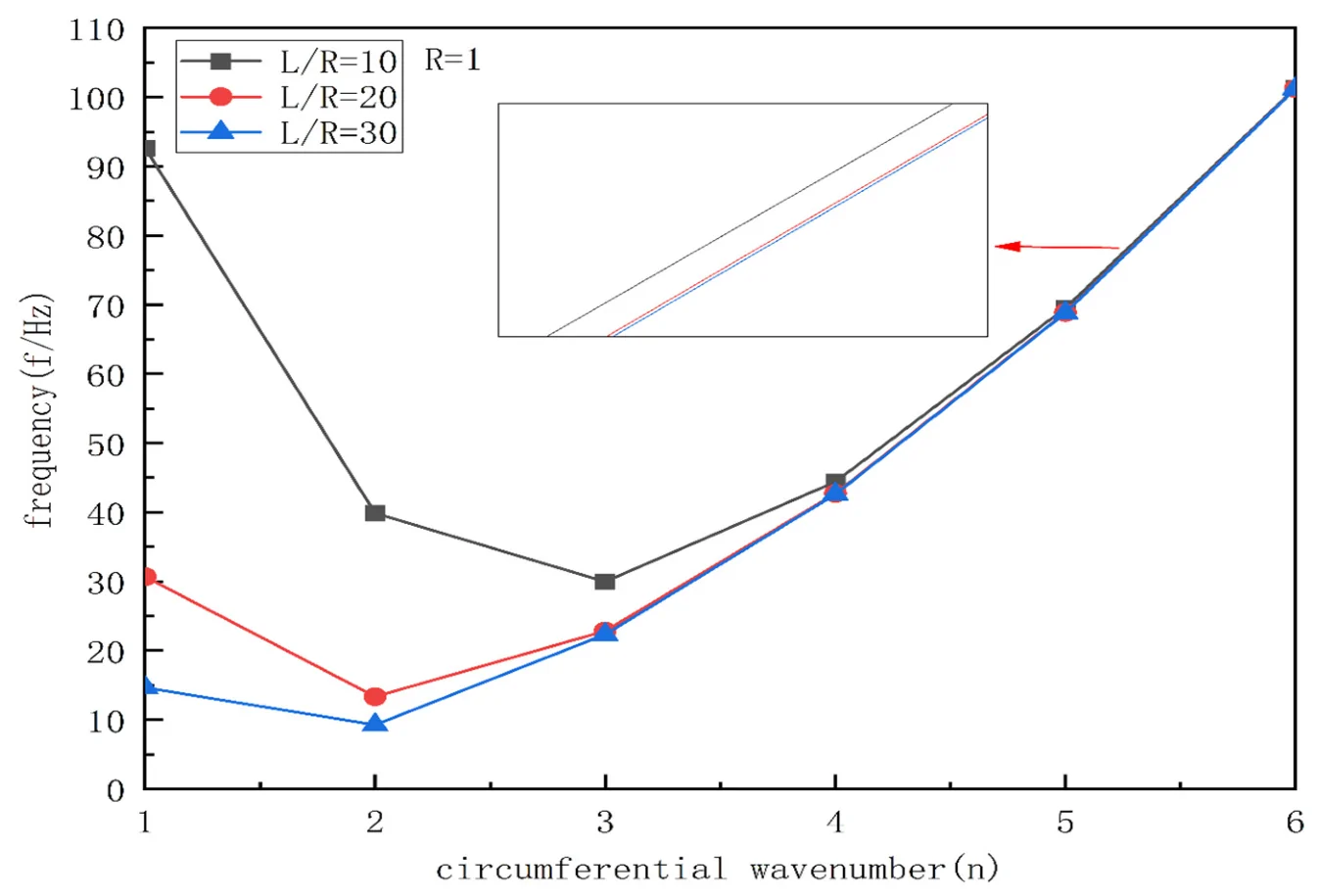
Frequency versus circumferential wavenumber for different length-to-radius ratios.

For the C-C boundary condition, with a shell radius of *R* = 1 and a varying shell length *L*, the vibration frequencies decrease as the length-to-radius ratio increases across different circumferential wavenumbers *n* (see Figure 11). The vibration frequency shows high sensitivity to the length-to-radius ratio for low-order vibration modes *n* ≤ 3, while for high-order modes *n* ≥ 4, the sensitivity is low or the frequency is not even affected. For lower modes with *n* ≤ 2, the vibration frequencies decrease with increasing circumferential wavenumber *n*, and the attenuation of the magnitude progressively lessens as the length-to-radius ratio increases.

### 6.4. Effect of Foundation Parameters

The vibration characteristics of cylindrical shells are investigated in this section with respect to the parameters of the visco-Pasternak foundation. Unless otherwise specified, the foundation parameters are set to $k_{w}$ = 100 MN/m^3^, $k_{s}$ = 10 MN/m, and $c_{d}$ = 0 [38].

The influence of foundation parameters on the vibration frequencies of cylindrical shells under various boundary conditions is shown in Table 5. It can be seen from Table 5 that, regardless of the boundary conditions, both the elastic modulus kw and the shear modulus ks of the elastic foundation significantly increase the vibration frequencies of the cylindrical shell, and the enhancement effect of kw is stronger than that of ks. When kw and ks act together, the total stiffness of the system increases further, resulting in a superimposed enhancement of the vibration frequency. However, the total frequency increase under their combined action does not exceed the sum of the increases when kw and ks act separately, which conforms to the linear superposition law based on the total system stiffness rather than a synergistic enhancement effect of “1 + 1 > 2”. Based on this physical law, under the premise of meeting frequency requirements, cost optimization can be achieved by properly matching the values of kw and ks. In addition, the enhancement effects of the foundation parameters kw and ks on the vibration frequencies of cylindrical shells under weakly constrained boundary conditions (C-F, S-S) are more significant than those under strongly constrained boundary conditions (C-C, C-S), indicating that weakly constrained boundaries are more sensitive to foundation parameters. This is mainly because strongly constrained boundaries themselves provide relatively large structural stiffness, leaving limited room for the additional stiffness contributed by foundation parameters, whereas weakly constrained structures are generally more flexible, and the additional stiffness introduced by the foundation support accounts for a larger proportion of the total system stiffness, thus leading to a more pronounced frequency increase. It can also be seen from Table 5 that, compared with the significant influence of the foundation parameters kw and ks, the influence of the graphene distribution type on the vibration frequencies of cylindrical shells is negligible and can be ignored.

**Figure 12:**
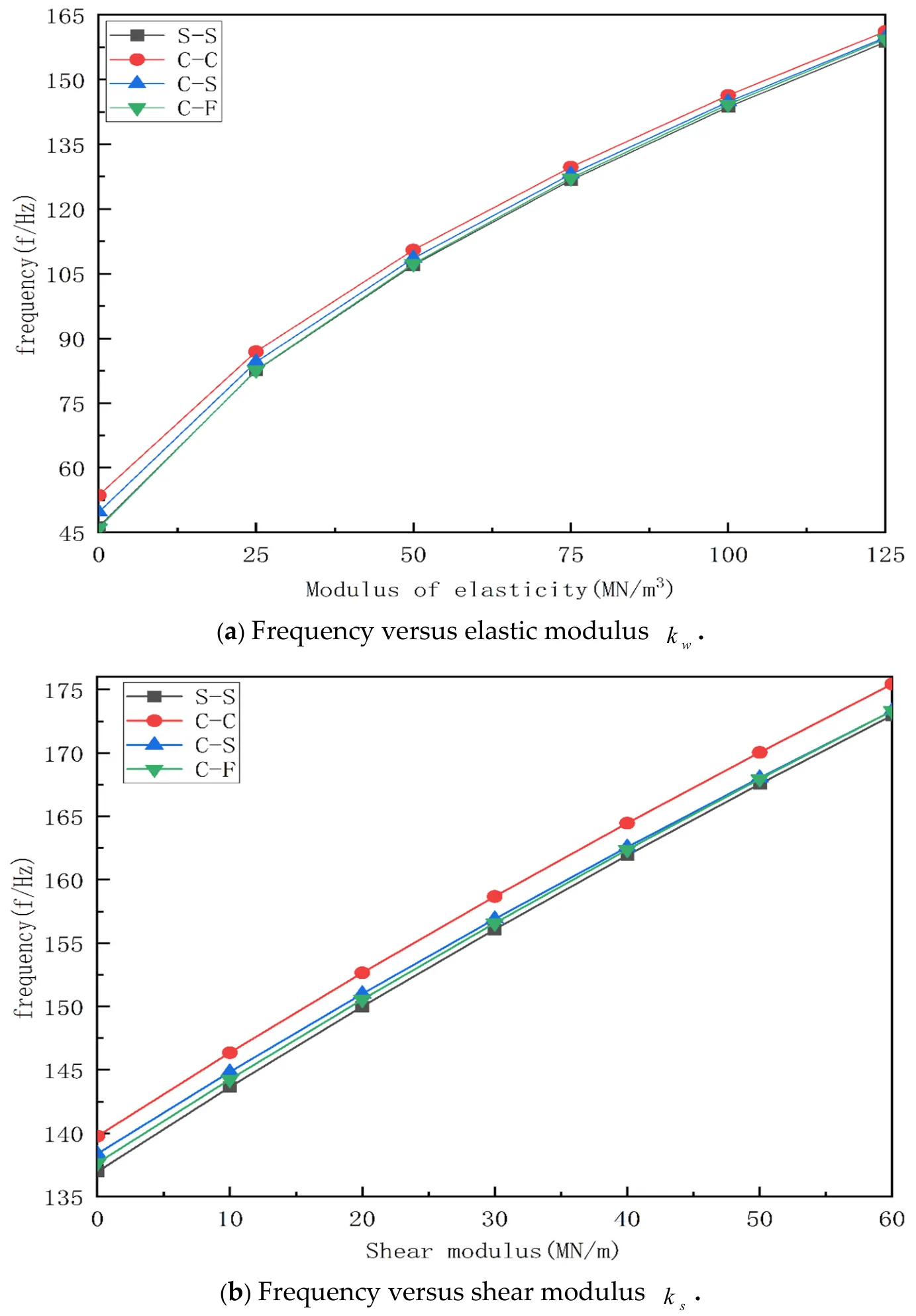
Variation in frequency with foundation parameters for different boundary conditions.

As is evident from Figure 12, the vibration frequencies for the four boundary conditions demonstrate a monotonic increase as the elastic modulus $k_{w}$ and shear modulus $k_{s}$ escalate, showing no decline or turning point. Notably, the rate of this escalation diminishes progressively with successive increments in the moduli. The influence of $k_{w}$ on the frequency is larger than that of $k_{s}$, and the boundary conditions do not alter the trend of the parameter effects, but only change the magnitude of the frequency values. In engineering practice, for soft soil foundations, priority can be given to enhancing $k_{w}$ through replacement and compaction, thereby rapidly improving the structural vibration resistance.

**Figure 13:**
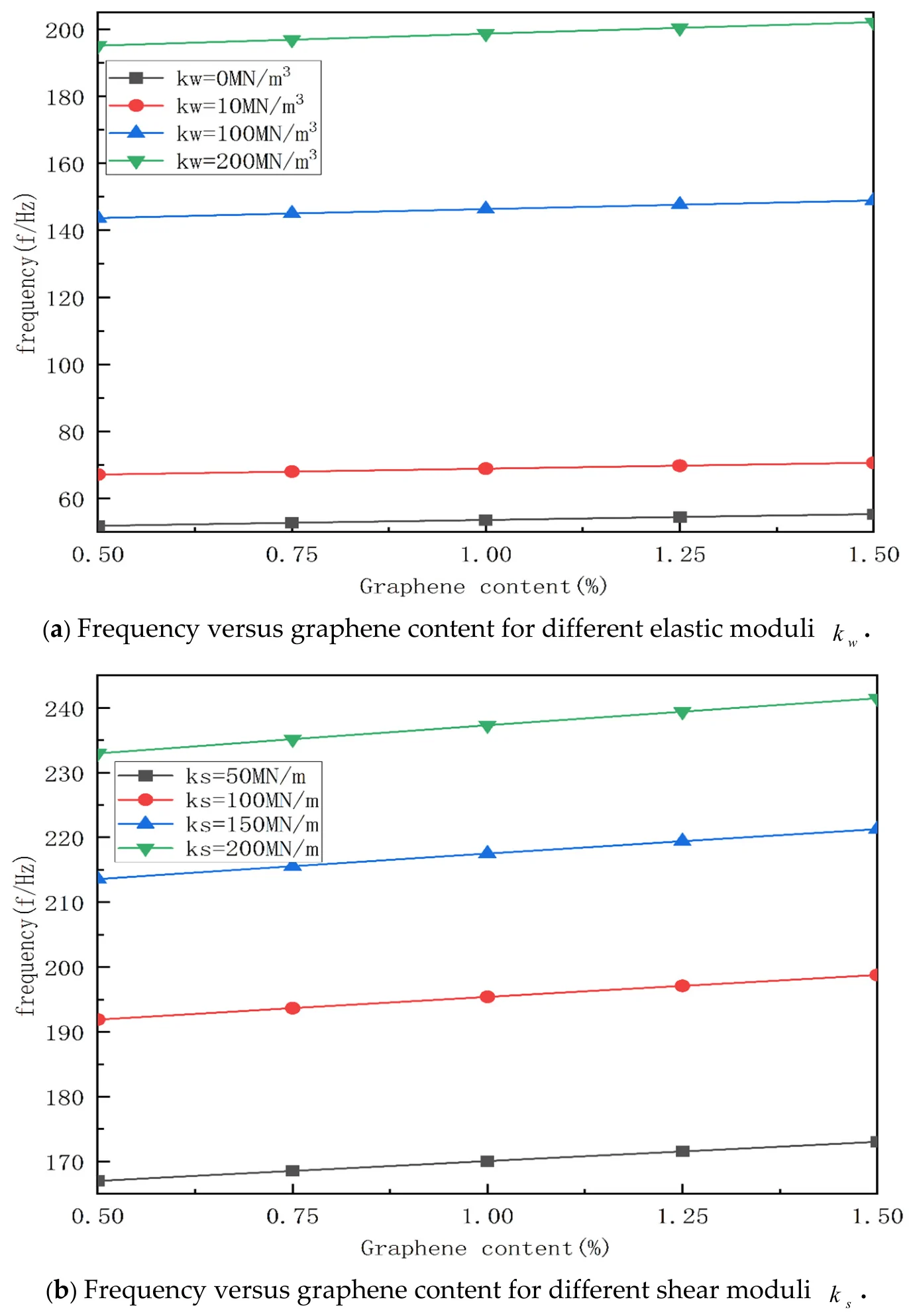
Variation in frequency with graphene content for different foundation parameters.

Figure 13 analyzes the effects of graphene content and foundation parameters on the vibration frequencies of cylindrical shells under C-C boundary conditions. As can be seen from the figure, because the value ranges of the elastic modulus kw and the shear modulus ks are much wider than that of the graphene content, they cause larger absolute changes in the vibration frequency within their respective ranges. Therefore, in practical engineering, kw and ks can serve as effective means for tuning the frequency over a wide range. The graphene content is limited by its relatively narrow conventional variation range, so its absolute adjustment range for the frequency is relatively limited; however, it still exhibits a stable enhancement effect per unit change and does not alter the increasing trend of the vibration frequency with respect to kw and ks. At high kw or high ks, the graphene content still maintains its enhancement effect. In engineering design, if a large change in the vibration frequency is required, adjusting kw or ks is more effective, whereas the graphene content can be used as an auxiliary tuning parameter for fine regulation.

**Figure 14:**
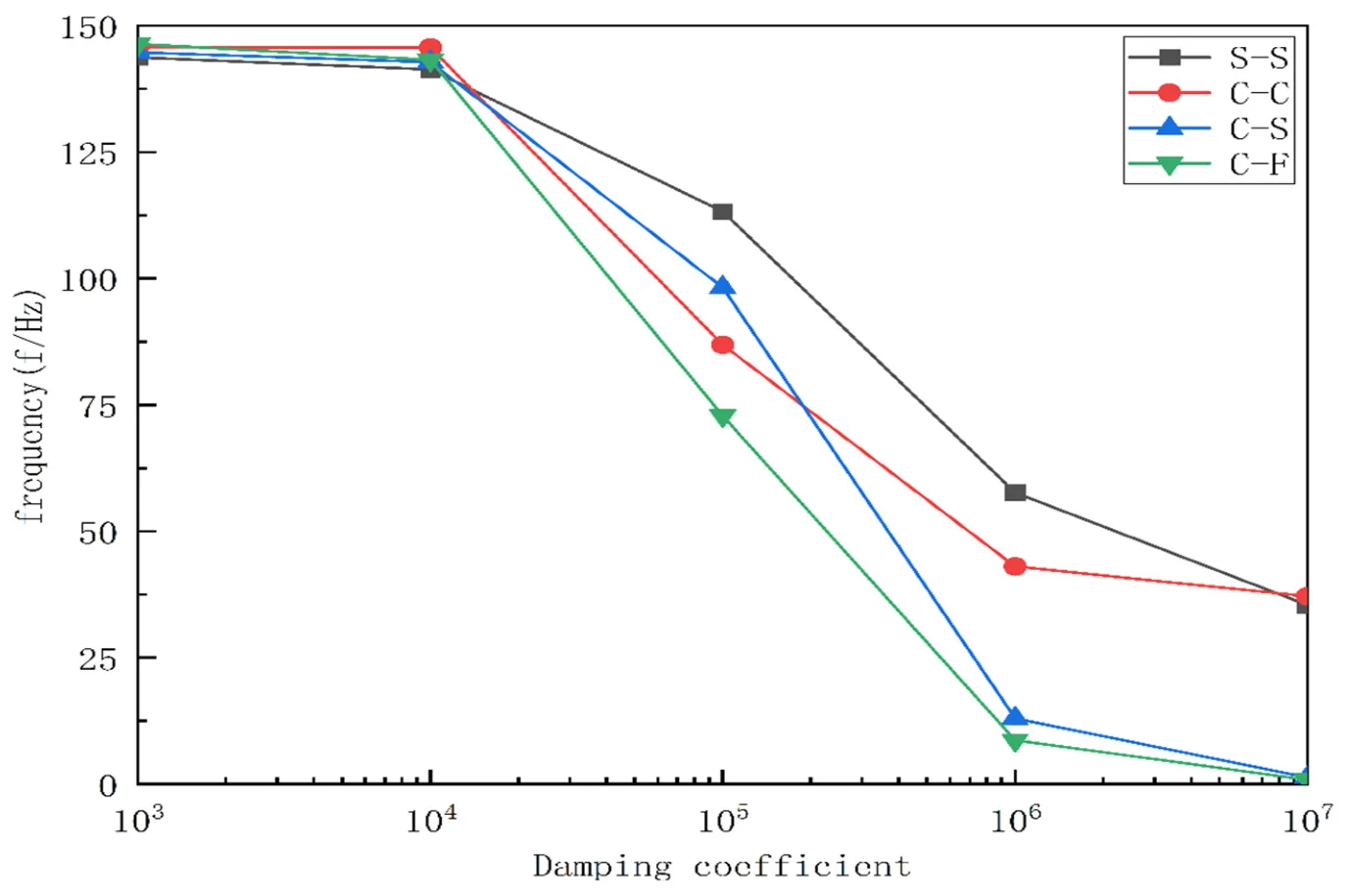
Variation in frequency with damping coefficient for different boundary conditions.

Figure 14 illustrates the sensitivity of frequency to the damping coefficient across different boundary conditions. Notably, the vibration frequencies diminish as the damping coefficient $c_{d}$ rises, confirming that damping exerts a suppressive effect on the frequency. However, the decline is not uniform but exhibits a stage-wise behavior. In the low-damping region, the frequencies fluctuate slightly and stay stable for all four boundary conditions. In the medium-damping region, frequencies drop quickly, particularly as $c_{d}$ approaches 10^5^. In the high-damping region, boundary effects diverge: C-F and C-S frequencies drop to nearly zero for $c_{d}$ ≥ 10^6^, while S-S and C-C retain frequencies of about 36 Hz. Since weakly constrained ends are more damping-sensitive, using C-S or C-F with a high-damping foundation is better for vibration suppression. For maintaining performance under a strong-damping foundation, C-C or S-S boundaries are preferable.

**Figure 15:**
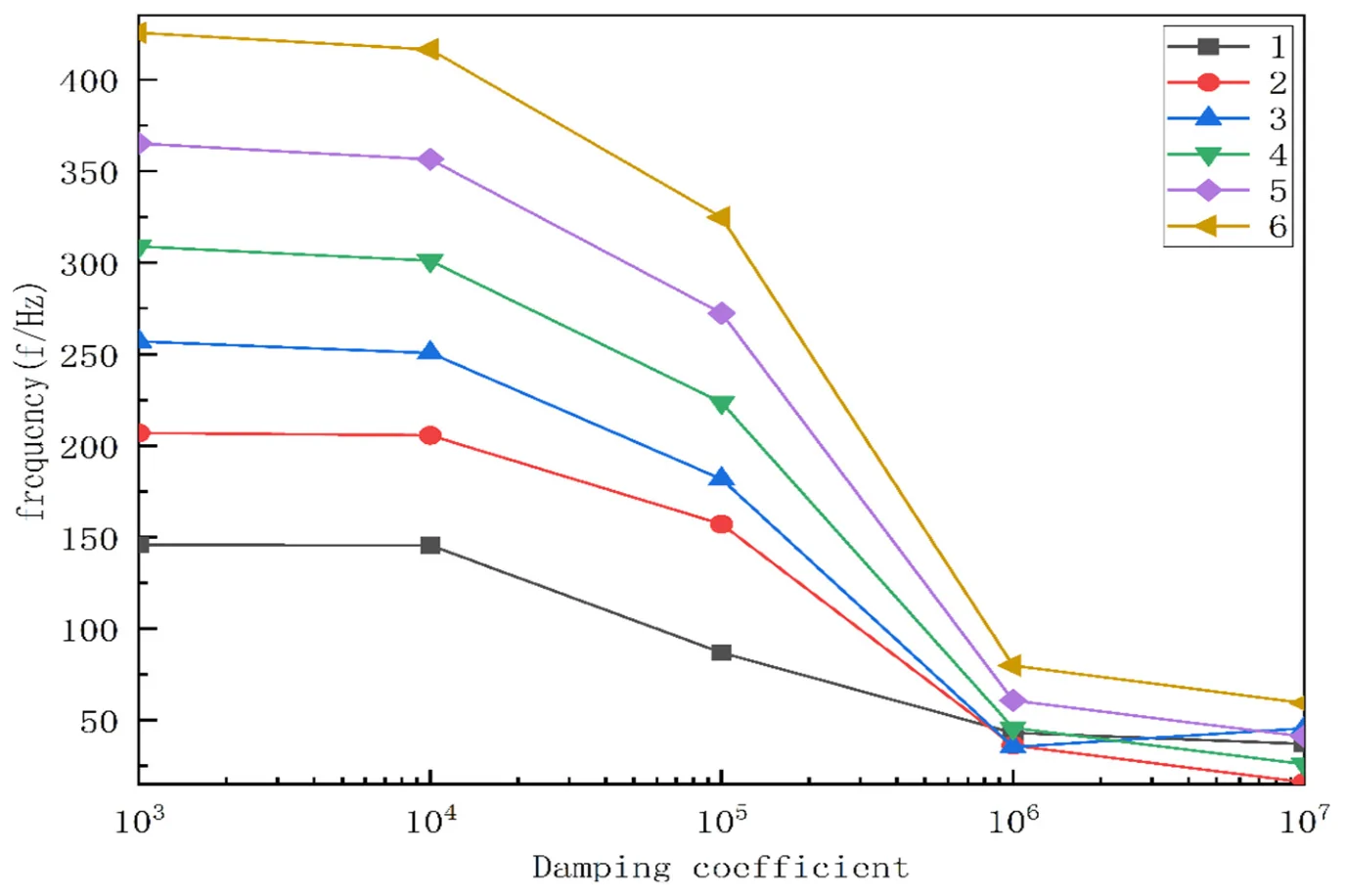
Variation in frequency with damping coefficient for different circumferential wavenumbers.

Figure 15 illustrates how the vibration frequency responds to the damping coefficient for various circumferential wavenumbers under the C-C boundary condition. From Figure 15, it can be observed that the vibration frequency decreases with increasing damping coefficient. When $c_{d}$ is below 10^4^, the damping has a minimal effect on the frequency, and the system exhibits a nearly undamped elastic response. Once $c_{d}$ exceeds 10^5^, the damping begins to dominate the dynamic characteristics of the system, and the frequency drops sharply. Under high damping, different circumferential wavenumbers exhibit varying sensitivities to the damping coefficient. Most notably, the frequency corresponding to *n* = 2 is the most sensitive to damping variations, and nearly vanishes under strong damping. In engineering practice, if the vibration frequency of specific modes is to be manipulated through the viscoelastic foundation, priority should be given to the design targeting highly sensitive modes (e.g., *n* = 2), as the vibrational energy of these modes is more readily dissipated by damping. In contrast, lower-order modes such as *n* = 1 require larger damping coefficients to achieve a significant frequency suppression effect.

## 7. Conclusions

This paper considers the interaction between porous materials, graphene reinforcement, and the visco-Pasternak foundation. Derived from the first-order shear deformation theory and Hamilton’s variational principle, the governing equations of vibration for functionally graded graphene-reinforced porous cylindrical shells were formulated. Natural frequencies under various boundary conditions were computed utilizing the differential quadrature method (DQM). Furthermore, a comprehensive parametric analysis was performed to assess the impacts of boundary conditions, material parameters, geometric parameters, and visco-Pasternak foundation parameters on the vibrational characteristics. The major findings are summarized as follows:

(1) Under the four typical constraints, the vibration frequency reaches a nadir before recovering as the circumferential wavenumber ascends, with the C-C condition consistently yielding the maximum value. The sensitivity of the vibration frequency to the boundary conditions diminishes with increasing circumferential wavenumber.

(2) Regardless of the boundary conditions, the elastic modulus $k_{w}$ and shear modulus $k_{s}$ of the foundation both significantly increase the vibration frequency, with the elastic modulus $k_{w}$ exhibiting a stronger enhancement effect than the shear modulus $k_{s}$. Weakly constrained boundaries are more sensitive to variations in the foundation parameters.

(3) As the graphene content increases, so does the vibration frequency. Among the three graphene distribution patterns, F-X yields the highest vibration frequency and possesses the greatest structural stiffness. Conversely, the vibration frequency decreases with the increasing porosity coefficient. The vibration frequency is predominantly governed by the elastic moduli ($k_{w}$ and $k_{s}$) of the foundation rather than the graphene content.

(4) Not only does the vibration frequency rise with the width-to-thickness ratio, but it also grows with the length-to-width ratio, with the former playing a dominant role. Beyond a width-to-thickness ratio of 1000, the vibration frequency levels off, and the impact of both the width-to-thickness ratio and the length-to-width ratio diminishes.

(5) The vibration frequency exhibits an inverse relationship with the radius-to-thickness ratio. When *n* ≤ 2, the frequency decreases with increasing circumferential wavenumber, with a relatively small variation amplitude, and the vibration frequency is relatively insensitive to the radius-to-thickness ratio. When *n* ≥ 3, the frequency increases with increasing circumferential wavenumber, with a large variation amplitude, and the vibration frequency exhibits a strong dependence on the radius-to-thickness ratio.

(6) The frequency shows an inverse trend with the length-to-radius ratio. For low-order modes (*n* ≤ 3), the vibration frequency is strongly dependent on this ratio, whereas the dependency weakens for high-order modes (*n* ≥ 4).

(7) Damping suppresses the frequency, but the frequency decreases non-uniformly and exhibits a stage-wise behavior. Specifically, in the low-damping region, the frequency fluctuates slightly and remains essentially stable, showing no obvious decline, and the frequencies obtained under the four boundary conditions are comparable. As the system transitions into the medium-damping region, the frequency declines sharply while damping starts to assert its influence. Conversely, in the high-damping region, the gaps among boundary conditions widen significantly.

(8) At high damping levels, the dependence of the natural frequencies on the damping coefficient varies with the circumferential wavenumber. The frequency corresponding to *n* = 2 is particularly sensitive and tends to vanish almost completely under strong damping. Consequently, when the viscoelastic foundation is used to adjust the vibration of selected modes, design efforts should focus on highly sensitive modes (e.g., *n* = 2), as their vibrational energy can be more effectively dissipated by damping.

The theoretical results of this study can provide references for the dynamic characteristic analysis and preliminary design of typical geotechnical and structural engineering problems, such as the natural frequency evaluation and resonance avoidance of underground utility tunnels, shield tunnel linings, deeply buried water/oil/gas pipelines, and large liquid storage tanks. Meanwhile, the theoretical solutions obtained from the linear free vibration model can also serve as reliable computational benchmarks for the development of numerical simulations and model validation of related novel composite structures.

## Figures and Tables

**Table 1:** Physical and mechanical properties of materials.

Material Properties	Steel	Graphene Platelets
Modulus of elasticity/GPa	200	1010
Density/kg/m^3^	7850	1062
Poisson’s ratio	0.34	0.186

**Table 2:** Comparison of natural frequencies of cylindrical shells with literature.

(*m*,*n*)	Natural Frequency (Hz)			Error (%)	
	Ref. [31]	Ref. [37]	Present	Difference with Ref. [31]	Difference with Ref. [37]
(1,3)	5.45	5.17	5.18	0.052	0.2
(1,4)	9.30	9.02	9.04	0.029	0.2
(1,5)	14.74	14.46	14.49	0.017	2.3
(2,3)	10.60	10.04	10.05	0.055	0.1
(2,4)	10.66	10.34	10.35	0.030	0.1
(2,5)	15.15	14.85	14.88	0.018	0.2
(3,3)	21.39	20.26	20.25	0.056	0.0
(3,4)	15.05	14.60	14.60	0.031	0.0
(3,5)	16.67	16.34	16.37	0.018	0.2

Note: The relative error (RD) in Table 2 is: $RD=\left|\frac{f_{ref.}-f_{present}}{f_{present}}\right|\times 100\%$, round the calculation results to the nearest whole number.

**Table 3:** Comparison of exact and numerical solutions.

(*m*,*n*)	Natural Frequency	
	Exact Solution	Semi-Analytic Solution (DQM)
(1,3)	5.18	5.1795
(1,4)	9.04	9.0378
(1,5)	14.49	14.4823

**Table 4:** Vibration frequencies of cylindrical shells under varying boundary conditions and distribution patterns.

Boundary Conditions	Patterns of Graphene Distribution	Frequency/Hz			
		$W_{G}$ = 0.75%	$W_{G}$ = 1%	$W_{G}$ = 1.25%	$W_{G}$ = 1.5%
S-S	F-X	14.4901	14.9984	15.4904	15.9666
	F-O	14.4895	14.9948	15.4870	15.9631
	F-U	14.4876	14.9953	15.4870	15.9634
C-C	F-X	29.6366	30.6797	31.6898	32.6677
	F-O	29.6350	30.6727	31.6825	32.6592
	F-U	29.6315	30.6727	31.6825	32.6583
C-S	F-X	23.5381	24.4185	25.2771	26.1154
	F-O	23.5266	24.3924	25.2426	26.0689
	F-U	23.5177	24.3843	25.2426	26.0500
C-F	F-X	14.0750	14.5692	15.1197	15.5104
	F-O	14.0745	14.5660	15.0444	15.5071
	F-U	14.0728	14.5662	15.0444	15.5073

**Table 5:** Vibration frequencies of cylindrical shells under different boundary conditions and with different foundation parameters.

Boundary Conditions	Foundation Parameters ($k_{w}$,$k_{s}$)	Frequency/Hz		
		F-X	F-O	F-U
S-S	(0,0)	14.9984	14.9984	14.9953
	(100,0)	136.9644	136.9622	136.9617
	(0,10)	46.3208	46.3190	46.3189
	(100,10)	143.6380	143.6356	143.6351
C-C	(0,0)	30.6797	30.6727	30.6727
	(100,0)	139.7684	139.7653	139.7649
	(0,10)	53.6128	53.6082	53.6080
	(100,10)	146.3613	146.3579	146.3576
C-S	(0,0)	24.4185	24.3924	24.3843
	(100,0)	138.3812	138.3747	138.3726
	(0,10)	49.7348	49.7214	49.7173
	(100,10)	144.8351	144.8286	144.8267
C-F	(0,0)	14.5692	14.5660	14.5662
	(100,0)	137.6485	137.6469	137.6466
	(0,10)	46.0152	46.0136	46.0134
	(100,10)	144.2501	144.2483	144.2480

## Data Availability

The original contributions presented in this study are included in the article. Further inquiries can be directed to the corresponding author.

## Appendix A

The manner in which each boundary condition is incorporated is as follows:

$$\begin{array}{l} A_{11}\sum_{j=1}^{M}g_{ij}^{\left(2\right)}U_{j}-[\frac{n^{2}A_{66}}{R^{2}}-I_{0}\omega^{2}]U_{i}-\frac{n}{R}[A_{12}+A_{66}]\sum_{j=1}^{M}g_{ij}^{\left(1\right)}V_{j}+\frac{A_{12}}{R}\sum_{j=1}^{M}g_{ij}^{\left(1\right)}W_{j}+\\ B_{11}\sum_{j=1}^{M}g_{ij}^{\left(2\right)}X_{j}-[\frac{n^{2}B_{66}}{R^{2}}-I_{1}\omega^{2}]X_{i}-\frac{n}{R}[B_{12}+B_{66}]\sum_{j=1}^{M}g_{ij}^{\left(1\right)}y_{j}=0 \end{array}$$

C-S

$$\begin{array}{c} A_{11}\sum_{j=1}^{M}g_{ij}^{\left(2\right)}U_{j}-[\frac{n^{2}A_{66}}{R^{2}}-I_{0}\omega^{2}]U_{i}\;\left(i=2,\;3\ldots \ldots \ldots M\right)\\ -\frac{n}{R}[A_{12}+A_{66}]\sum_{j=1}^{M}g_{ij}^{\left(1\right)}V_{j}\;\left(i=2,\;3\ldots \ldots \ldots M-1\right)\\ \frac{A_{12}}{R}\sum_{j=1}^{M}g_{ij}^{\left(1\right)}W_{j}\;\left(i=2,\;3\ldots \ldots \ldots M-1\right)\\ B_{11}\sum_{j=1}^{M}g_{ij}^{\left(2\right)}X_{j}-[\frac{n^{2}B_{66}}{R^{2}}-I_{1}\omega^{2}]X_{i}\;\left(i=2,\;3\ldots \ldots \ldots M\right)\\ -\frac{n}{R}[B_{12}+B_{66}]\sum_{j=1}^{M}g_{ij}^{\left(1\right)}Y_{i}\;\left(i=2,\;3\ldots \ldots \ldots M-1\right) \end{array}$$

C-F

$$\begin{array}{c} A_{11}\sum_{j=1}^{M}g_{ij}^{\left(2\right)}U_{j}-[\frac{n^{2}A_{66}}{R^{2}}-I_{0}\omega^{2}]U_{i}\;\left(i=2,\;3\ldots \ldots \ldots M\right)\\ -\frac{n}{R}[A_{12}+A_{66}]\sum_{j=1}^{M}g_{ij}^{\left(1\right)}V_{j}\;\left(i=2,\;3\ldots \ldots \ldots M\right)\\ \frac{A_{12}}{R}\sum_{j=1}^{M}g_{ij}^{\left(1\right)}W_{j}\;\left(i=2,\;3\ldots \ldots \ldots M\right)\\ B_{11}\sum_{j=1}^{M}g_{ij}^{\left(2\right)}X_{j}-[\frac{n^{2}B_{66}}{R^{2}}-I_{1}\omega^{2}]X_{i}\;\left(i=2,\;3\ldots \ldots \ldots M\right)\\ -\frac{n}{R}[B_{12}+B_{66}]\sum_{j=1}^{M}g_{ij}^{\left(1\right)}Y_{i}\;\left(i=2,\;3\ldots \ldots \ldots M\right) \end{array}$$

## References

[1] Akinwande D., Brennan C.J., Bunch J.S., Egberts P., Felts J.R., Gao H., Huang R., Kim J.-S., Li T., Li Y. (2017). A review on mechanics and mechanical properties of 2D materials—Graphene and beyond. Extrem. Mech. Lett..

[2] Zhao S., Zhao Z., Yang Z., Ke L.-L., Kitipornchai S., Yang J. (2020). Functionally graded graphene reinforced composite structures: A review. Eng. Struct..

[3] Boggarapu V., Gujjala R., Ojha S., Acharya S.K., Babu P.V., Chowdary S., Gara D.K. (2021). State of the art in functionally graded materials. Compos. Struct..

[4] Xu F., Zhang X., Zhang H. (2018). A review on functionally graded structures and materials for energy absorption. Eng. Struct..

[5] Betts C. (2012). Benefits of metal foams and developments in modelling techniques to assess their materials behaviour: A review. Mater. Sci. Technol..

[6] Dong Y.H., Li Y.H., Chen D., Yang J. (2018). Vibration characteristics of functionally graded graphene reinforced porous nanocomposite cylindrical shells with spinning motion. Compos. Part B Eng..

[7] Wang Y., Gu Y., Li H., Han B. (2016). Research on traveling wave resonance characteristics of high-speed rotating thin-walled cylindrical shells. J. Vib. Shock.

[8] Wang Y.Q., Guo X.H., Chang H.H., Ba Y. (2009). Nonlinear vibration characteristics of mode precession of rotating thin-walled cylindrical shells. Acta Mech. Solida Sin..

[9] Lazreg H., Mehmet A., Nafissa Z. (2022). Natural frequency analysis of imperfect FG sandwich plates resting on Winkler–Pasternak foundation. Mater. Today Proc..

[10] Nosier A., Fallah F. (2009). Non-linear analysis of functionally graded circular plates under asymmetric transverse loading. Int. J. Non-Linear Mech..

[11] Ye T., Jin G., Chen Y., Liu H. (2013). Free vibration analysis of laminated composite shallow shells with general elastic boundaries. Compos. Struct..

[12] Li Y.T., Liu X.N., Hu Z.Q., Kan Y.T., Li B.Q. (2025). Research on surface-similarity scaled boundary finite element method for plate and shell structures based on symplectic system. Eng. Mech..

[13] Zuo P., Shi X.J. (2024). Vibration characteristics of functionally graded conical shell-annular plate under temperature field. J. Harbin Eng. Univ..

[14] Huang X.L., Li L.J., Zhang Y.N., Hao X.Q. (2023). Vibration of porous graphene-reinforced composite truncated conical shells in elastic medium. J. Henan Univ. Sci. Technol. (Nat. Sci. Ed.).

[15] Huang X.L., Wang C.Z., Wei N.G., Xiao W.W. (2024). Flutter and buckling analysis of graphene-reinforced porous functionally graded truncated conical shells. J. Kunming Univ. Sci. Technol. (Nat. Sci. Ed.).

[16] Gao Z.Y., Shi X.J. (2025). Dynamic modeling and characteristic analysis of functionally graded graphene-reinforced composite conical shells. J. Vib. Eng..

[17] Huang X.L., Zhang Y.N., Wei N.G., Li L.J. (2023). Vibration and buckling study of porous carbon nanotube-reinforced composite truncated conical shells. J. Henan Polytech. Univ. (Nat. Sci. Ed.).

[18] Zhang Y., Wang Y., Li H. (2023). Vibration characteristics of functionally graded graphene-reinforced thin-walled cylindrical shells with elastic boundary. J. Univ. Sci. Technol. Liaoning.

[19] Zhang F. (2021). Vibration and stability of graphene sheet-reinforced nanocomposite micro cylindrical shells. Sci. Technol. Eng..

[20] Zhang J.H., Liu G. (2025). Analytical solution for free vibration of graphene platelet-reinforced functionally graded cylindrical shells. Chin. J. Comput. Mech..

[21] Wang Y., Xu Z.Q., Li H., Wang P., Xu H.D., Zhang Y. (2024). Vibration characteristics of functionally graded graphene-reinforced spinning cylindrical shells. J. Henan Polytech. Univ. (Nat. Sci. Ed.).

[22] Jian Z., Wang Y., Wang P., Li H., Li X.H. (2024). Vibration characteristics analysis of functionally graded graphene-reinforced stepped cylindrical shells. Noise Vib. Control.

[23] Sofiyev A., Hui D., Haciyev V., Kadioglu F., Erdem D., Yuan G.Q., Schnack E. (2017). The nonlinear vibration of orthotropic functionally graded cylindrical shells surrounded by an elastic foundation within first order shear deformation theory. Compos. Part B Eng..

[24] Shao D. (2017). Analysis of Dynamic Performances for Composite Laminated Structures Subjected to Arbitrary Boundary and Coupling Conditions. Ph.D. Thesis.

[25] Gao T., Zheng H.Y. (2024). Nonlinear vibration study of functionally graded three-phase composite cylindrical shells. J. Dyn. Control.

[26] Li Y.Q., Guo X.H., Yan S.Y. (2001). Wave vibration response analysis of cylindrical shells. Chin. J. Appl. Mech..

[27] Dong Y.H., Li Y.H., Li X.Y., Mao X.C. (2024). Research on vibration characteristics of spinning cylindrical shells under multiple boundary conditions. J. Vib. Eng..

[28] Sharma C.B. (1974). Calculation of natural frequencies of fixed-free circular cylindrical shells. J. Sound Vib..

[29] Kiarasi F., Babaei M., Sarvi P., Asemi K., Hosseini M., Omidi Bidgoli M. (2021). A review on functionally graded porous structures reinforced by graphene platelets. J. Comput. Appl. Mech..

[30] Li Q., Wu D., Chen X., Liu L., Yu Y., Gao W. (2018). Nonlinear vibration and dynamic buckling analyses of sandwich functionally graded porous plate with graphene platelet reinforcement resting on Winkler–Pasternak elastic foundation. Int. J. Mech. Sci..

[31] Wang Q.Y., Ye C., Zu J.W. (2018). Nonlinear vibration of metal foam cylindrical shells reinforced with graphene platelets. Aerosp. Sci. Technol..

[32] Afshari H. (2022). Free vibration analysis of GNP-reinforced truncated conical shells with different boundary conditions. Aust. J. Mech. Eng..

[33] Cong F., Zhang R., Li W., Jin Y., Yu G., Wu L. (2024). Buckling analysis of moderately thick carbon fiber composite cylindrical shells under hydrostatic pressure. Appl. Ocean Res..

[34] Imran M., Shi D., Tong L., Elahi A., Uddin M. (2021). On the elastic buckling of cross-ply composite closed cylindrical shell under hydrostatic pressure. Ocean Eng..

[35] Xu H.D., Wang Y., Zhang Y., Jia X.Y., Li X.H. (2024). Vibration characteristics of graphene-reinforced porous cylindrical shells with arbitrary boundary conditions. Chin. J. Solid Mech..

[36] Shu C. (2000). Differential Quadrature and Its Application in Engineering.

[37] Chai Q., Qing Y.W. (2022). Traveling wave vibration of graphene platelet reinforced porous joined conical-cylindrical shells in a spinning motion. Eng. Struct..

[38] Safarpour M., Ghabussi A., Ebrahimi F., Habibi M., Safarpour H. (2020). Frequency characteristics of FG-GPLRC viscoelastic thick annular plate with the aid of GDQM. Thin-Walled Struct..

